# Tobit modeling for dependent-sample *t*-tests and moderated regression with ceiling or floor data

**DOI:** 10.3758/s13428-025-02904-y

**Published:** 2025-12-10

**Authors:** Lijuan Wang, Ruoxuan Li

**Affiliations:** Department of Psychology, University of Notre Dame, 390 Corbett Hall, Notre Dame, IN 46556 USA

**Keywords:** Ceiling effects, Floor effects, t test, Moderated regression, Tobit modeling

## Abstract

Ceiling or floor effects pose analytic challenges in behavioral and psychological research. In this study, we developed novel Tobit modeling approaches, estimated using maximum likelihood (ML) or Bayesian methods, to address these effects for widely used statistical analyses, including the dependent-sample *t*-test and moderated regression. Simulation studies were conducted to compare the performance of the proposed modeling approaches to the conventional approach where ceiling or floor data are treated as if true values. The conventional approach was found to yield biased estimates, inflated Type I error rates, and poor confidence interval coverage, even with as little as 10% ceiling data. In contrast, the proposed approaches with either ML or Bayesian estimation provided accurate estimates and inference results across most studied conditions (e.g., with 30% ceiling data). Real data examples further illustrated the impact of modeling choices. To facilitate implementations of the proposed Tobit modeling approaches, we provide simulated datasets along with R and Mplus scripts online. Implications of the findings and future research directions were discussed.

**Supplementary Information:**

The online version contains supplementary material available at 10.3758/s13428-025-02904-y.

## Introduction

Ceiling or floor effects occur when measurement scales are too easy or too difficult and thus a substantial proportion of individuals obtain the maximum or minimum score (Uttl, 2005). These effects are prevalent in behavioral, psychological, and medical research. For example, Uttl (2005) found ceiling effects in 25% or more of 9- to 15-item verbal list learning tasks, and in 50% or more of the verbal paired-associations learning tasks used to assess memory in cognitive psychology. In social surveys, ceiling effects often appear in ’top-coding’ questions (Austin & Brunner, 2003), such as those asking about income, where the highest category represents an income greater than a certain threshold. Similarly, in medical research, ceiling effects have been observed in measurement scales like pain assessment tools used to evaluate outcomes in hip replacement surgery (Lim et al., 2015). Some researchers argued that the use of sum scores from measurement scales with too easy or too difficult Likert items can lead to ceiling or floor effects (Edwards & Soland, 2024; McNeish, 2024). For individuals with ceiling or floor effects, their true scores are not observed. Specifically, when a true score exceeds the maximum score (or the ceiling threshold), the observed sum score is the maximum score. Similarly, when a true score is smaller than the minimum score (or the floor threshold), the observed sum score is the minimum score.

When ceiling or floor effects occur, there are mass points with dense frequencies at the maximum or the minimum in the distribution of observed data. Note that observed data from a semicontinuous variable ((Olsen & Schafer, 2001)) also have mass points – for example, alcohol or other substance usage often has a mass point at zero. In learning research, performance asymptotes (Miller, 1956) lead to distributions with mass points as well, such as at the upper and/or lower asymptotic values. Wang et al. (2008) noted that the mass point value in a semicontinuous variable (e.g., zero alcohol usage) is a true data value. Likewise, performance asymptote values are the greatest or lowest true values participants can demonstrate and thus are also true data values. In the current study, we follow the definition in Uttl (2005) and assume ceiling and floor effects occur before participants reach asymptotic values, making the mass point values from ceiling and floor effects proxies for larger or smaller true values. Consequently, statistical methods for handling ceiling or floor effects should be different from the two-part models used for semicontinuous variables (e.g., Olsen and Schafer, 2001) and models developed to data analyses with performance asymptotes (e.g., Feng et al., 2019; Singer and Willett, 2003).

Liu and Wang (2021) conducted a literature review to study how psychological and educational researchers statistically handled ceiling or floor effects in independent-sample *t*-tests and ANOVA. They found that over 57% of the reviewed articles using *t*-tests and 70% of those using ANOVA ignored ceiling or floor effects, treating ceiling or floor data as if there were true scores in the analyses. In this study, we refer to this practice as the conventional approach due to its widespread use. The conventional approach underestimates marginal means in the presence of ceiling effects and overestimates them in the presence of floor effects. When both ceiling and floor effects occur, the extent to which observed means deviate from true means depends on the proportions of ceiling and floor data. Marginal variances are also underestimated under this approach. Researchers have demonstrated that the conventional approach may produce misleading statistical inference results under the presence of ceiling data (Liu & Wang, 2021; Wang et al., 2008). For instance, Liu and Wang (2021) observed biased estimates and inflated Type I error rates in independent-sample *t*-tests and ANOVA when ceiling effects existed in the outcomes. Similarly, Austin and Brunner (2003) found inflated Type I error rates in multiple regression analyses when ceiling effects existed in a predictor.

In biostatistics or economics, censored data arise when the value of an observation is only partially known due to certain restrictions. For example, in biostatistics, right-censored data occur when a patient’s survival time is only known to exceed a certain point (e.g., 5 years) because they were still alive at the end of the study. Ceiling and floor data share similar statistical properties of right- or left-censored data in biostatistics and economics. As a result, Tobit modeling, introduced by Tobin (1958) to handle limited or censored data in economics, has been adapted to address ceiling and floor effects across various models and methods in psychology and behavioral research (Liu & Wang, 2021; Muthen, 1990; Wang et al., 2008; Wang & Zhang, 2011).

When applying Tobit modeling to handle ceiling or floor effects, the key idea is to specify different likelihood functions for data with and without these effects. For example, when the underlying distribution is normal, the normal density function is used to specify the likelihoods for data without ceiling or floor effects, whereas the cumulative density function is used to specify the likelihoods for data with ceiling or floor effects. Maximum likelihood (ML) estimation has been employed to estimate Tobit models for addressing censored data in factor analysis (Muthen, 1989) and for handling ceiling or floor data in multiple regression (Austin & Brunner, 2003), longitudinal data analysis (Twisk & Rijmen, 2009), and independent-sample *t* tests and ANOVA (Liu & Wang, 2021). Additionally, Bayesian methods have been developed to address ceiling or floor effects in latent growth curve modeling (Wang et al., 2008) and mediation analysis (Wang & Zhang, 2011). Research has consistently shown that Tobit modeling, using either ML or Bayesian methods, outperforms the conventional approach when analyzing censored or ceiling/floor data. There is considerable discussion in the literature regarding the advantages of using Bayesian estimation over ML estimation, such as its ability to incorporate prior information and estimate complex random-effects or latent variable models (e.g., Du et al., 2017; McNeish, 2016; Zhang et al., 2008). However, no prior research has systematically compared the performance of Tobit modeling using ML estimation versus Bayesian estimation for handling ceiling or floor effects.

To our knowledge, no prior research has developed or evaluated methods to address ceiling or floor effects for the dependent-sample *t*-test, a widely used statistical test. Although extending Tobit modeling from the independent-sample *t*-test to the dependent-sample *t*-test may seem straightforward, it presents practical challenges (to be discussed in the next section). Furthermore, in most existing studies, Tobit models have been developed to address ceiling or floor effects in dependent variables (e.g., outcomes, mediators). An exception is the work by Austin and Brunner (2003), who developed a likelihood function for multiple regression with two predictors, one of which exhibits ceiling effects. However, their approach is applicable to their studied multiple regression model and does not extend to more complex scenarios, such as multiple regression with moderators. Moreover, the likelihood function they proposed can be challenging for applied researchers to implement. For example, in moderated regression where the regression model involves moderators and thus interaction terms, the interaction terms are subject to ceiling or floor effects when their corresponding focal predictors and/or moderators are subject to these effects. However, how to statistically address these effects in interaction terms has not been studied previously.

To fill in the research gaps, we developed novel modeling approaches integrating both Tobit modeling and latent variable modeling to handle ceiling or floor effects for two widely used statistical techniques: the dependent-sample *t*-test and moderated regression. In the proposed Tobit models, ceiling or floor effects in both the pre-test and post-test scores for the dependent-sample *t*-test and in both the outcome variable and predictors/moderators for moderated regression can be accounted for. For both the dependent-sample *t*-test and moderated regression, ML and Bayesian methods were developed to estimate the proposed Tobit models and implemented in Mplus and R JAGS, respectively. Furthermore, we conducted Monte Carlo simulation studies to evaluate and compare the performance of the proposed Tobit modeling approaches to that of the conventional approach as well as compare the performance of ML vs. Bayesian estimation for Tobit modeling. Real data analysis examples and simulated data with Mplus and R JAGS scripts were provided to illustrate the modeling approaches for both the dependent-sample *t*-test and moderated regression.

In the remainder of the article, we will first introduce our proposed Tobit modeling approaches with ML and Bayesian estimation for the dependent-sample *t*-test. Then we will describe the design and results of a simulation study to evaluate the performance of different modeling approaches for handling ceiling or floor effects in dependent-sample *t*-tests, followed by a real data analysis example. After that, for moderated regression, we will present our proposed Tobit modeling approaches with ML and Bayesian estimation, describe the design and results from a separate simulation study for performance evaluation, and then a real data analysis example. Finally, we will conclude the article by discussing the implications of our findings and suggesting directions for future research.

## The dependent-sample *t*-test with ceiling or floor data

The dependent-sample *t*-test (also known as the paired-sample *t*-test) is widely used to compare the means of two related samples, such as pre-test and post-test measurements or matched-pairs samples. For illustrative purposes, we consider the pre-test and post-test scenario as an example to introduce the modeling approaches. In such a research setting, researchers have observed data from individual *i* $(i=1,2,..,N)$ at time point *t* ($t=$ 1 for pre-test and $t=$ 2 for post-test), denoted as $y_{it}$. When ceiling or floor effects occur, the true scores $y_{it}^{*}$ may not be fully observed. Specifically, when a true score $y_{it}^{*}$ falls below or equals the minimum score *a* (the floor threshold), the observed score $y_{it}$ is constrained at *a*. When a true score $y_{it}^{*}$ exceeds the maximum score *b* (the ceiling threshold), the observed score $y_{it}$ is capped at *b*. For individuals without ceiling or floor effects, their true scores are observed directly and fall within the range of *a* and *b*. Together, the observed scores $y_{it}$ can be expressed as:

1 $$\begin{aligned} y_{it}={\left\{ \begin{array}{ll} a & y_{it}^{*}\le a\\ y_{it}^{*} & a<y_{it}^{*}<b\\ b & y_{it}^{*}\ge b \end{array}\right. }. \end{aligned}$$

In the conventional dependent-sample *t* test where ceiling or floor effects are ignored, observed scores rather than true scores are used.

The *t*-test statistic is:

2 $$\begin{aligned} t=\frac{\bar{y}_{2}-\bar{y}_{1}}{\sqrt{\frac{1}{N}(s_{1}^{2}+s_{2}^{2}-2s_{12})}}, \end{aligned}$$

where $\bar{y}_{1}$ and $\bar{y}_{2}$ are the sample means of the pre-test and post-test observed scores, *N* is the sample size, $s_{1}^{2}$ and $s_{2}^{2}$ are the sample variances of the pre-test and post-test observed scores, and $s_{12}$ is the sample covariance between the pre-test and post-test observed scores. Under the null hypothesis, the *t*-statistic follows a *t* distribution with $N-1$ degrees of freedom.

Cohen’s *d*, a commonly used effect size measure for this test, can be calculated as:

3 $$\begin{aligned} d=\frac{\bar{y}_{2}-\bar{y}_{1}}{\sqrt{(s_{1}^{2}+s_{2}^{2}-2s_{12})}}=\dfrac{t}{\sqrt{N}}. \end{aligned}$$

To address ceiling or floor effects, we propose Tobit modeling approaches with ML or Bayesian estimation, implemented in popular software programs Mplus and R JAGS, respectively.

### Tobit modeling approach with ML estimation

As reviewed in the introduction, the key idea of Tobit modeling is to use different likelihood functions for data with or without ceiling or floor effects. In this study, we extend Tobit modeling to the dependent-sample *t*-test, accounting for ceiling or floor effects. For the observed data, the likelihood functions are based on either the normal density or the cumulative normal distribution, assuming that the true scores follow a normal distribution. Such likelihood functions have been implemented in R packages ’censReg’ and ’survival’ for Tobit or censored regression analysis with ML estimation. However, these packages were developed for handling a single censored outcome variable. Thus, they cannot be directly used for the dependent-sample *t*-test with ceiling or floor effects. Mplus provides a function for modeling censored data, in which the Tobit modeling idea is applied and ML estimation is used (Muthen, 1990). However, this implementation has a limitation: it does not allow covariances among censored variables, nor between censored and other observed variables. This poses a challenge for the dependent-sample *t*-test with ceiling or floor effects, as the covariance between the two dependent samples is nonzero and must be accounted for.

To address the limitations, we integrated Tobit modeling with latent variable modeling, enabling the application of Tobit modeling to the dependent-sample *t*-test with ceiling and floor effects. We used Mplus to implement the approach as it has existing functions for both censored data and latent variable modeling. Specifically, we use the censored data specification in Mplus to separately identify ceiling or floor data in $Y_{1}$ and $Y_{2}$ as in Eq. 1. Then, we model the true scores $y_{it}^{*}$ in Eq. 1 using a common factor model as below:

4 $$\begin{aligned} \begin{array}{c} y_{i1}^{*}=\alpha _{1}+\lambda _{1}\eta _{i}+\epsilon _{i1}\\ y_{i2}^{*}=\alpha _{2}+\lambda _{2}\eta _{i}+\epsilon _{i2} \end{array}, \end{aligned}$$

where the common factor $\eta$ as well as the unique factors $\epsilon _{1}$ and $\epsilon _{2}$ follow independent normal distributions. To identify the model, we set the mean of $\eta$ to be 0 and its variance to be 1, while constraining the means of the unique factors ($\epsilon _{1}$ for pre-test and $\epsilon _{2}$ for post-test) to 0 and their variances to be equal ($\sigma _{e}^{2}$). The two intercepts ($\alpha _{1}$ and $\alpha _{2}$) and the two factor loadings ($\lambda _{1}$ and $\lambda _{2}$) are freely estimated.

With this model specification, $\alpha _{1}$ and $\alpha _{2}$ represent the means of the pre-test and post-test true scores, respectively. The covariance between the pre-test and post-test true scores is allowed and modeled through the common factor $\eta$. The model-implied covariance matrix of the pre-test and post-test true scores is

5 $$\begin{aligned} \Sigma =\left[ \begin{array}{cc} \lambda _{1}^{2}+\sigma _{e}^{2}\\ \lambda _{1}\lambda _{2} & \lambda _{2}^{2}+\sigma _{e}^{2} \end{array}\right] . \end{aligned}$$

In the Tobit model with ML estimation for the dependent-sample *t*-test, the likelihood function of the observed score $y_{it}$ is specified as:

6 $$\begin{aligned} l_{it}={\left\{ \begin{array}{ll} \Upphi (\frac{a-\alpha _{t}}{\sqrt{\lambda _{t}^{2}+\sigma _{e}^{2}}}) & \textrm{if}\,y_{it}=a\\ \phi (\frac{y_{it}-\alpha _{t}}{\sqrt{\lambda _{t}^{2}+\sigma _{e}^{2}}}) & \mathrm {if\,}a<y_{it}<b\\ \mathrm {1-}\Upphi (\frac{b-\alpha _{t}}{\sqrt{\lambda _{t}^{2}+\sigma _{e}^{2}}}) & \mathrm {if\,}y_{it}=b \end{array}\right. }, \end{aligned}$$

where $\phi (x)$ and $\Upphi (x)$ represent the standard normal density function and the standard normal cumulative density function, respectively. When an observed datum does not have floor or ceiling effects, the standard normal density function is used for the likelihood function (as shown in the middle line of Eq. 6). However, when an observed datum has floor or ceiling effects, the standard normal cumulative distribution function is used instead (as shown in the first or third lines of Eq. 6, respectively). The likelihood function of the model is

7 $$\begin{aligned} L=\prod _{i}\intop _{\eta _{i}}\prod _{t}l_{it}N(\eta _{i};0,1)d\eta _{i}. \end{aligned}$$

Under this Tobit approach, the mean difference estimate is $\hat{\alpha _{2}}-\hat{\alpha }_{1}$ and the corresponding *t*-test statistic can be constructed as

8 $$\begin{aligned} t=\frac{\hat{\alpha _{2}}-\hat{\alpha }_{1}}{\sqrt{\left( \hat{\lambda }_{1}^{2}+\hat{\lambda }_{2}^{2}+2\hat{\sigma _{e}^{2}}-2\hat{\lambda }_{1}\hat{\lambda }_{2}\right) /N}} \end{aligned}$$

A central *t* distribution with $N-1$ degrees of freedom is used as the reference distribution to obtain the *p* value and confidence interval estimates. Note that in the default Mplus output, however, the standard normal distribution is used as the reference distribution for the *p* value and confidence interval estimation. We will evaluate the performance of the proposed *t*-test as well as the robustness of using the two reference distributions in a simulation study, which will be presented below. Cohen’s *d* can be calculated by $t/\sqrt{N}$ by substituting the *t*-value from Eq. 8.

### Tobit modeling approach with Bayesian estimation

The dependent-sample *t*-test can also be written with the true scores in the latent variable modeling framework, in a more intuitive way, as below:

9 $$\begin{aligned} \begin{array}{c} y_{i1}^{*}=\eta _{1i}+\epsilon _{1i}\\ y_{i2}^{*}=\eta _{1i}+\eta _{2i}+\epsilon _{2i} \end{array}, \end{aligned}$$

where pre-test and post-test true scores $y_{1}^{*}$ and $y_{2}^{*}$ are modeled using two latent variables $\eta _{1}$ and $\eta _{2}$. $\eta _{1}$ and $\eta _{2}$ follow independent normal distributions: $\eta _{1}\sim N(\mu _{1},\sigma _{1}^{2})$ and $\eta _{2}\sim N(\mu _{D},\sigma _{D}^{2})$, with $\mu _{1}$ representing the mean of the pre-test true scores and $\mu _{D}$ denoting the mean difference between pre-test and post-test true scores. The errors $\epsilon _{1}$ and $\epsilon _{2}$ are assumed to follow independent normal distributions with zero means and equal variances $\sigma _{e}^{2}$. The equality constraint on the error variances ($\sigma _{e}^{2}$ ) is imposed for model identification.

Under this model specification, the correlation between the pre-test and post-test true scores is modeled via the common latent variable $\eta _{1}$. The model-implied covariance matrix of the pre-test and post-test true scores is:

10 $$\begin{aligned} \Sigma =\left[ \begin{array}{cc} \sigma _{1}^{2}+\sigma _{e}^{2}\\ \sigma _{1}^{2} & \sigma _{1}^{2}+\sigma _{D}^{2}+\sigma _{e}^{2} \end{array}\right] . \end{aligned}$$

In this Tobit Bayesian approach for the dependent-sample *t*-test, the likelihood function of the observed score $y_{it}$ is:

11 $$\begin{aligned} l_{i1}&={\left\{ \begin{array}{ll} \Upphi (\frac{a-\mu _{1}}{\sqrt{\sigma _{1}^{2}+\sigma _{e}^{2}}}) & \textrm{if}\,y_{i1}=a\\ \phi (\frac{y_{i1}-\mu _{1}}{\sqrt{\sigma _{1}^{2}+\sigma _{e}^{2}}}) & \textrm{if}\,a<y_{i1}<b\\ \mathrm {1-}\Upphi (\frac{b-\mu _{1}}{\sqrt{\sigma _{1}^{2}+\sigma _{e}^{2}}}) & \textrm{if}\,y_{i1}=b \end{array}\right. }\mathrm {\qquad and\qquad }\nonumber \\ l_{i2}&={\left\{ \begin{array}{ll} \Upphi (\frac{a-\mu _{1}-\mu _{D}}{\sqrt{\sigma _{1}^{2}+\sigma _{D}^{2}+\sigma _{e}^{2}}}) & \textrm{if}\,y_{i2}=a\\ \phi (\frac{y_{i2}-\mu _{1}-\mu _{D}}{\sqrt{\sigma _{1}^{2}+\sigma _{D}^{2}+\sigma _{e}^{2}}}) & \textrm{if}\,a<y_{i2}<b\\ \mathrm {1-}\Upphi (\frac{b-\mu _{1}-\mu _{D}}{\sqrt{\sigma _{1}^{2}+\sigma _{D}^{2}+\sigma _{e}^{2}}}) & \textrm{if}\,y_{i2}=b \end{array}\right. } \end{aligned}$$

The likelihood function of the model can be written as:

12 $$\begin{aligned} L=\prod _{i}\intop _{\boldsymbol{\eta }_{i}}\prod _{t}l_{it}N(\mathbf {\boldsymbol{\eta }}_{i};\boldsymbol{0},\boldsymbol{D})d\boldsymbol{\eta }_{i}, \end{aligned}$$

where $$\mathbf {\boldsymbol{\eta }}_{i}=(\eta _{1i},\eta _{2i})$$ and $$\mathbf {\boldsymbol{\eta }}_{i}\sim N(0,\boldsymbol{D}=\left[ \begin{array}{cc} \sigma _{1}^{2}\\ \sigma _{1}^{2} & \sigma _{1}^{2}+\sigma _{D}^{2} \end{array}\right] )$$.

To implement Bayesian estimation for the model, we used R JAGS, in which the censored function in JAGS (i.e., ’dinterval’) is used to separately identify ceiling or floor effects in the pre-test and post-test scores as in Eq. 1. As described above, the covariance of the pre-test and post-test true scores is allowed and modeled via the common latent variable $\eta _{1}$.

In addition to specifying the likelihood function, prior distributions for the model parameters need to be specified. For this model, noninformative priors can be used. Specifically, in this study, priors for the mean parameters ($\mu _{1}$ and $\mu _{D}$) were $N(0,10^{6})$, and the priors for the variance parameters ($\sigma _{1}^{2}$, $\sigma _{D}^{2}$, and $\sigma _{e}^{2}$) were *Gamma*(0.001, 0.001). Monte Carlo Markov chain (MCMC) sampling methods, such as Gibbs sampling, can be used to generate the posterior distributions of the model parameters. From the posterior distributions, point estimates of the model parameters can be obtained, for example, using the posterior means. Statistical inference can be made using 95% percentile credible intervals.

Under this model, Cohen’s *d* at the population level is:

13 $$\begin{aligned} \frac{\mu _{D}}{\sqrt{\sigma _{D}^{2}+2\sigma _{e}^{2}}}. \end{aligned}$$

The 95% percentile credible intervals of Cohen’s *d* can be used to make inferences about the effect size measure.

Although we used two different model specifications (Eqs. 4 and 9) for ML and Bayesian estimation, the two proposed Tobit models are mathematically equivalent. Specifically, the error variance $\sigma _{e}^{2}$ is identical in the two model specifications. Additionally, we have $\alpha _{1}=\mu _{1}$, $\alpha _{2}=\mu _{1}+\mu _{D}$, $\lambda _{1}^{2}=\sigma _{1}^{2}$, $\lambda _{2}^{2}=\sigma _{1}^{2}+\sigma _{D}^{2}$, and $\lambda _{1}\lambda _{2}=\sigma _{1}^{2}$ to link the parameters in the two model specifications.

### A simulation study

We conducted a simulation study to evaluate the performance of the three approaches for addressing ceiling/floor effects in the dependent-sample *t*-test: the conventional approach, the proposed Tobit approach with ML estimation (Tobit ML), and the proposed Tobit approach with Bayesian estimation (Tobit Bayesian). We focused on the scenario of having ceiling data as an example, and the findings can be generalized to scenarios with floor data when the true population distribution is symmetrical (Liu & Wang, 2021).

**Table 1:** The dependent-sample *t*-test simulation results: Empirical bias and relative bias in mean difference estimates from conditions with $\rho =0.5$

		CP = 0%	CP = 10%			CP = 20%			CP = 30%		
				Tobit	Tobit		Tobit	Tobit		Tobit	Tobit
N	SDR	Reference	Conventional	ML	Bayesian	Conventional	ML	Bayesian	Conventional	ML	Bayesian
Empirical bias under Cohen’s $d=0$											
50	1	.012	.009	.008	.014	-.001	.001	.013	-.003	-.004	.019
50	1.5	.000	**-.115**	.004	.003	**-.165**	-.006	-.009	**-.183**	-.005	-.014
100	1	.002	.000	.001	.004	-.005	-.004	.003	-.003	-.004	.011
100	1.5	.000	**-.116**	-.001	.003	**-.160**	-.003	.000	**-.182**	-.004	-.003
200	1	.001	.000	.000	.003	.000	.001	.006	-.001	.000	.009
200	1.5	-.001	**-.118**	-.004	.000	**-.153**	.005	.008	**-.182**	.000	.006
500	1	-.002	-.003	-.003	-.001	-.001	-.001	.003	-.002	-.002	.004
500	1.5	.000	**-.117**	-.003	.000	**-.158**	-.002	.002	**-.181**	.000	.004
Relative bias under Cohen’s $d=0.5$											
50	1	-.011	**-.139**	.017	.022	**-.277**	.003	.030	**-.382**	.024	.077
50	1.5	-.011	**-.442**	.006	-.011	**-.607**	-.004	-.022	**-.721**	.016	-.027
100	1	-.003	**-.153**	.003	.011	**-.275**	.006	.028	**-.394**	.000	.041
100	1.5	.003	**-.442**	.001	.003	**-.608**	-.001	.001	**-.719**	.008	-.004
200	1	.003	**-.151**	.002	.013	**-.274**	.009	.023	**-.387**	.010	.039
200	1.5	.001	**-.441**	.001	.009	**-.608**	-.004	.008	**-.727**	-.006	.007
500	1	-.003	**-.155**	.000	.005	**-.281**	-.002	.007	**-.394**	.000	.016
500	1.5	.000	**-.443**	-.003	.001	**-.608**	-.004	.004	**-.725**	-.003	.008

Note. CP: ceiling proportion of pre-test scores; SDR: the population standard deviation ratio. Relative bias higher than 10% was highlighted in bold

#### Simulation design

In this simulation study, we considered five factors: Cohen’s *d* ($d=$ 0 and 0.5, corresponding to null and medium size effects), the population standard deviation ratio of $y_{2}^{*}$ to $y_{1}^{*}$ ($SDR=$ 1 and 1.5, representing homogeneous and heterogeneous post-test to pre-test standard deviation ratios), the correlation between $y_{2}^{*}$ and $y_{1}^{*}$ ($\rho =$ 0 and 0.5, representing no correlation and high correlation between pre-test and post-test true scores^1^), the ceiling proportion of the pretest scores ($CP=$ 0%, 10%, 20%, and 30%), and the sample size ($N=$ 50, 100, 200, and 500). In total, there were $2\times 2\times 2\times 4\times 4=128$ conditions with 1000 replications for each condition.

True scores at pre-test and post-tests ($y_{1}^{*}$ and $y_{2}^{*}$) were generated from normal distributions: $y_{1}^{*}\sim N(0,1)$ and $y_{2}^{*}\sim N(\mu _{2},SDR^{2})$. The true mean of $y_{2}^{*}$ was determined by $\mu _{2}=d\sqrt{(1+SDR^{2}-2*\rho *SDR)}$. To generate ceiling data, the ceiling threshold was first determined by applying $1-CP$ to the inverse cumulative normal density function. This ceiling threshold was then used to create observed data for both the pre-test and post-test ($y_{1}$ and $y_{2}$). The data were generated in $\text {R}$ software. The conventional, Tobit ML, and Tobit Bayesian approaches were implemented in $\text {R}$, $\text {Mplus}$, and $\text {R JAGS}$, respectively. In each replication of the Bayesian analysis, two Markov chains were used, each with a burn-in period of 30,000 iterations and 60,000 iterations after-burn-in iterations for the posterior distributions. Convergence was assessed using the potential scale reduction factor (PSRF) from the Gelman-Rubin convergence diagnostic. A PSRF value greater than 1.1 for any model parameter indicates nonconvergence, suggesting the need for more iterations (Gelman & Rubin, 1992). In this study, convergence was claimed when all parameters had their PSRF values below 1.1 and nonconvergence was counted if any parameter had a PSRF above 1.1. A 90% or higher convergence rate was viewed as satisfactory.

The performance of the three modeling approaches was further evaluated based on the estimation accuracy and coverage of the mean difference using estimates from converged replications. Estimation accuracy was assessed using empirical bias when the population value was null, and using relative bias when the population value was nonzero. A relative bias greater than 10% was considered non-ignorable (Muthén & Muthén, 2002). Coverage was evaluated by the empirical Type I error rates when the population value was null and by the 95% confidence/credible interval coverage rates when the population value was nonzero. Empirical Type I error rates between 2.5% to 7.5% were viewed as satisfactory (Bradley, 1978), and coverage rates between 91% to 98% were considered acceptable (Muthén & Muthén, 2002). Note that coverage rates are functions of both estimation accuracy (how close a parameter estimate is to the true value) and estimation precision (uncertainty or variability in the parameter estimate). Therefore, coverage rates are related to estimation accuracy such that coverage rates are likely to be poor if the estimation accuracy is low.

**Table 2:** The dependent-sample *t*-test simulation results: Empirical Type I error rates and coverage rates of the mean difference from conditions with $\rho =0.5$

		CP = 0%	CP = 10%			CP = 20%			CP = 30%		
				Tobit	Tobit		Tobit	Tobit		Tobit	Tobit
N	SDR	Reference	Conventional	ML	Bayesian	Conventional	ML	Bayesian	Conventional	ML	Bayesian
Empirical Type I error rates (%) under Cohen’s $d=0$											
50	1	5.4	5.1	5.6	3.8	6.2	5.5	5.0	4.3	5.2	4.3
50	1.5	5.8	**9.7**	5.0	4.1	**17.5**	5.6	5.1	**23.4**	5.1	5.2
100	1	5.3	4.2	4.4	3.8	4.7	5.7	4.9	4.7	5.6	4.7
100	1.5	5.0	**18.2**	5.9	5.4	**31.9**	5.2	4.9	**46.0**	5.4	5.6
200	1	4.7	5.0	4.9	5.1	4.6	4.9	4.9	5.2	5.2	5.0
200	1.5	5.8	**30.8**	4.9	5.0	**52.3**	3.8	3.7	**76.2**	4.8	4.6
500	1	4.2	5.7	5.9	5.2	4.3	4.0	4.1	4.2	4.6	4.9
500	1.5	4.7	**63.3**	4.4	4.4	**91.9**	4.9	5.1	**99.2**	5.6	5.9
Coverage rates (%) under Cohen’s $d=0.5$											
50	1	95.6	**90.6**	93.6	95.1	**77.1**	94.8	95.6	**54.4**	94.7	95.3
50	1.5	94.2	**50.0**	93.9	95.2	**15.6**	94.7	94.1	**2.1**	95.6	95.4
100	1	94.6	**87.1**	96.3	96.0	**58.7**	95.0	94.2	**24.3**	94.7	95.3
100	1.5	95.4	**20.0**	94.5	94.7	**1.1**	95.2	95.4	**0.0**	94.5	94.3
200	1	94.9	**76.8**	94.7	94.8	**34.4**	95.0	95.4	**3.2**	94.7	95.4
200	1.5	94.7	**1.5**	95.2	95.2	**0.0**	95.3	95.8	**0.0**	94.2	95.1
500	1	94.4	**49.1**	96.0	95.3	**3.8**	93.9	94.2	**0.0**	95.2	95.7
500	1.5	95.6	**0.0**	95.4	94.6	**0.0**	95.5	94.2	**0.0**	94.7	94.4

Note. CP: ceiling proportion of pre-test scores; SDR: the population standard deviation ratio. Empirical Type I error rates outside the 2.5–7.5% range and coverage rates outside the 91–98% range were highlighted in bold

#### Simulation results

Convergence rates are presented in the supplemental materials (Tables S1 and S2). The conventional and Tobit ML approaches both achieved 100% convergence rates, while the Tobit Bayesian approach had convergence rates ranging from 99.9% to 100%. The empirical bias and relative bias results from conditions with $\rho =0.5$ are shown in Table 1. The coverage rates and empirical Type I error rates from those conditions are presented in Table 2. Results from conditions with $\rho =0$ shared the same patterns as those from conditions with $\rho =0.5$ and are available in the online supplemental materials (Tables S3 and S4).

The proposed Tobit approaches, whether using ML or Bayesian estimation, had very small empirical bias or ignorable relative bias in all studied conditions. In contrast, the conventional approach showed larger empirical bias when the effect size was zero and nonignorable relative bias when the effect size was non-null. Empirical bias or relative bias from the conventional approach was higher when the ceiling proportions or heterogeneity in the variances (SDR) were greater. For example, under conditions with $N=100$, Cohen’s $d=0.5$, and $SDR=1.5$, the relative bias in the mean difference estimates was 44.2% when the ceiling proportion was 10%; the relative bias was increased to 71.9% when the ceiling proportion was 30% (see Table 1).

Similar patterns were observed in the empirical Type I error rates and coverage rates from the conventional approach, with more severe inflation of Type I error rates and lower coverage rates in cases where sample variances were heterogeneous ($SDR=1.5$) and ceiling proportions were higher. For example, with $N=100$ and $SDR=1.5$, the empirical Type I error rates were 18.2%, 31.9%, and 46.0% when the ceiling proportion was 10%, 20%, and 30%, respectively, and the coverage rates were 20.0%, 1.1%, and 0%, respectively. In contrast, the Tobit modeling approaches with ML or Bayesian estimation exhibited satisfactory empirical Type I error rates and coverage rates across all studied conditions. Note that the results from Tobit ML reported in Table 2 were directly from the outputs of Mplus, where the standard normal distribution was used as the reference distribution for statistical inference. We also computed the empirical Type I error rates and coverage rates based on the *t* distribution with $df=N-1$ for Tobit ML and these results are displayed in Table S5. As seen in Tables 2 and S5, using either inference approach, Tobit ML yielded satisfactory empirical Type I error rates and coverage rates across all studied conditions.

### An illustrative real data analysis example

We used a subset of the real data in Wang et al. (2008) collected by Salthouse (2004) to illustrate the impact of modeling choices on the dependent-sample *t*-test with ceiling effects. Ninety-six participants aged between 19 and 30 were measured on the Wechsler Memory Scale (WAIS) III Word Lists subtest across four occasions. At each occasion, a list of 12 unrelated words was presented to the participants followed immediately by an attempt to recall as many of the words as possible and this process was repeated for three times with the same words in the same order. In this example, we used data from the first time recall of the last two occasions (named as $Y_{1}$ and $Y_{2}$). We were interested in comparing the means of recall at these two occasions. Due to the practice effects, 32 (33.3%) and 40 (41.7%) participants had the maximum score (i.e., 12) in the recall tests at the two occasions, respectively, and thus ceiling effects were substantial in the data.

The observed means were 10.74 and 10.92 with observed variances at 1.51 and 1.51, respectively. When the conventional dependent-sample *t* test was conducted, the mean difference estimate was 0.18 (Cohen’s *d* = 0.17); the *t* statistic was 1.69 with $df=95$ and a *p* value of 0.094 (in the output of Mplus, the *p* value was .092 and was based on the standard normal distribution), indicating that the two means were not statistically significantly different. When the Tobit approach with ML estimation was used, however, the mean difference estimate was 0.38 (Cohen’s *d* = 0.26); the *t* statistic was 2.04 with $df=95$ and a *p* value of 0.045 (in the output of Mplus, the *p* value was .042 and was based on the standard normal distribution), indicating that the two means were statistically significantly different. When the Tobit approach with Bayesian estimation was used, the mean difference estimate (posterior mean) was 0.38 (95% percentile credible interval: 0.013, 0.759) and the posterior mean of Cohen’s *d* was 0.26 (95% percentile credible interval: 0.014, 0.510), indicating that the two means were statistically significantly different. Overall, findings from the Tobit approaches with ML or Bayesian estimation were consistent, whereas the statistical conclusion from the conventional approach was different. According to the simulation results, we recommend the use of the results from the Tobit modeling approaches.

To facilitate the implementation of the Tobit modeling approaches with ML or Bayesian estimation for the dependent-sample *t*-test, we shared a simulated data set from the simulation condition with Cohen’s $d=0.5$, $SDR=1$, $\rho =0.5$, pre-test $CP=30\%$, and $N=100$ as well as the Mplus and R JAGS scripts in the online Supplemental Materials.

## Moderated regression with ceiling or floor data

In psychological and behavioral research, researchers are often interested in testing whether the effect of a predictor variable on an outcome variable depends on the level of a third variable, known as a moderator (Baron & Kenny, 1986). Such analyses can provide valuable insights into for whom and under what conditions an effect is stronger or weaker, ultimately aiding in the development of intervention programs (e.g., Park et al., 2018). One of the most commonly used methods for moderation analysis is moderated regression (e.g., Hayes, 2013), which can be written as

14 $$\begin{aligned} y_{i}=\beta _{0}+\beta _{1}x_{i}+\beta _{2}z_{i}+\beta _{3}x_{i}z_{i}+e_{i} \end{aligned}$$

where $y_{i}$, $x_{i}$, and $z_{i}$ represent the observed outcome, focal predictor, and moderation scores for individual *i*, respectively, and $e_{i}$ is a residual term assumed to have a mean of zero and variance $\sigma _{e}^{2}$. In this model, regression coefficients $\beta _{1}$ and $\beta _{2}$ capture the main effects of the focal predictor and moderator on the outcome, respectively, and $\beta _{3}$ represents the moderation (interaction) effect. In addition, $\beta _{1}+\beta _{3}z$ quantifies the simple effect of the focal predictor on the outcome at a given value of *z*. In practice, researchers are often interested in estimating and testing the moderation effect $\beta _{3}$ and the simple effects $\beta _{1}+\beta _{3}z$ at representative values of *z*.

When ceiling or floor effects are present but ignored in conventional moderated regression analyses, the estimation accuracy and testing performance of moderation effects have not been systematically examined in prior research. This study aims to address this gap by investigating the impact of ceiling or floor effects on statistical inference of moderation effects. Because the means and variances of the observed scores from variables with ceiling or floor effects are biased estimates, we anticipate that the conventional approach may lead to misleading statistical inference regarding moderation effects.

In moderated regression, ceiling or floor effects can occur not only in the outcome variable but also in the focal predictor or moderator variables. For example, in a study examining the effect of pre-test working memory scores on post-test working memory scores, with vocabulary scores as a potential moderator, ceiling effects could arise in both the outcome (post-test working memory) and the focal predictor (pre-test working memory). As reviewed in the introduction, prior research on handling ceiling or floor effects in predictor variables is limited, with the exception of Austin and Brunner (2003). However, their proposed likelihood function is complex and not easily implementable or extendable (e.g., from regression with two predictors to moderated regression with an interaction term), making it less accessible for applied researchers.

In this section, we introduce practical Tobit modeling approaches to address ceiling or floor effects in predictor, moderator, and/or outcome variables for moderated regression. To facilitate the introduction, we discuss two situations: (1) only the outcome variable is subject to ceiling or floor effects, and (2) the outcome, predictor, and moderator variables are all subject to such effects. For the first situation, the Tobit model introduced by Tobin (1958) can be directly applied with ML or Bayesian estimation, which we review below. For the second situation, Bayesian estimation using software such as JAGS allows for a relatively straightforward application of Tobit modeling. However, for maximum likelihood (ML) estimation, existing programs such as Mplus and R packages ’censReg’ and ’survival’ do not readily accommodate Tobit modeling in this context. Therefore, we propose an ML-based Tobit modeling approach that integrates traditional Tobit modeling with latent variable techniques to effectively handle ceiling or floor effects in both outcome and predictor/moderator variables for moderated regression.

### Tobit moderated regression for outcomes with ceiling or floor effects

To handle ceiling effects that occur only in the outcome variable, the traditional Tobit approach can be directly applied. Instead of modeling the observed outcome scores ($y_{i}$), the analysis is conducted using true scores $y_{i}^{*}$, which are defined by:

15 $$\begin{aligned} y_{i}={\left\{ \begin{array}{ll} a_{Y} & y_{i}^{*}\le a_{Y}\\ y_{i}^{*} & a_{Y}<y_{i}^{*}<b_{Y}\\ b_{Y} & y_{i}^{*}\ge b_{Y} \end{array}\right. }. \end{aligned}$$

where $a_{Y}$ and $b_{Y}$ are the floor and ceiling thresholds for outcome variable *Y*, respectively. The Tobit moderated regression model can then be written as:

16 $$\begin{aligned} y_{i}^{*}=\beta _{0}+\beta _{1}x_{i}+\beta _{2}z_{i}+\beta _{3}x_{i}z_{i}+e_{i} \end{aligned}$$

Under the assumption that regression residuals ($e_{i}$) follow a normal distribution, the likelihood function of the observed outcome $y_{i}$ is specified as

17 $$\begin{aligned} l_{i}={\left\{ \begin{array}{ll} \Upphi (\frac{a_{Y}-\beta _{0}-\beta _{1}x_{i}-\beta _{2}z_{i}-\beta _{3}x_{i}z_{i}}{\sigma _{e}}) & \textrm{if}\,y_{i}=a_{Y}\\ \phi (\frac{y_{i}-\beta _{0}-\beta _{1}x_{i}-\beta _{2}z_{i}-\beta _{3}x_{i}z_{i}}{\sigma _{e}}) & \mathrm {if\,}a_{Y}<y_{i}<b_{Y}\\ \mathrm {1-}\Upphi (\frac{b_{Y}-\beta _{0}-\beta _{1}x_{i}-\beta _{2}z_{i}-\beta _{3}x_{i}z_{i}}{\sigma _{e}}) & \mathrm {if\,}y_{i}=b_{Y} \end{array}\right. }. \end{aligned}$$

This model can be estimated in Mplus and R packages ’censReg’ and ’survival’ using the censored data specification for *Y* with ML estimation. It can also be implemented in R JAGS with Bayesian estimation by specifying *Y* as a censored variable using the ’dinterval’ function. This allows for the likelihood function to be explicitly defined as shown above in Eq. 17. For Bayesian estimation, noninformative priors can be used to facilitate estimation. For example, the regression coefficients can have diffuse normal priors (e.g., $N(0,10^{6})$) and the residual variance can have a Gamma prior (e.g., *Gamma*(0.001, 0.001)).

### Tobit moderated regression for outcomes and predictors/moderators with ceiling or floor effects

#### With Bayesian estimation

Beyond the outcome variable, the censored distribution function implemented in R JAGS also provides a straightforward way to handle ceiling or floor effects for focal predictors and moderators. Suppose that the observed variables *y*, *x*, and *z* are all subject to ceiling or floor effects. In the proposed Tobit Bayesian modeling approach, each observed variable is first modeled using a separate censored normal distribution (through the function ’dinterval’), for constructing the true scores $y^{*}$, $x^{*}$, and $z^{*}$, respectively. The definitions of $x^{*}$ and $z^{*}$ follow the same strategy as for $y^{*}$ in Eq. 15, while different ceiling or floor thresholds can be applied to different variables. The interaction term $x_{i}^{*}z_{i}^{*}$ is constructed based on the product of $x^{*}$ and $z^{*}$. Thus, ceiling or floor effects are accounted for in the interaction term. The moderated regression analysis is then conducted using these true scores instead of the observed scores to estimate and test the regression coefficients. The Tobit moderated regression model under Bayesian estimation is specified as:

18 $$\begin{aligned} y_{i}^{*}=\beta _{0}+\beta _{1}x_{i}^{*}+\beta _{2}z_{i}^{*}+\beta _{3}x_{i}^{*}z_{i}^{*}+e_{i}. \end{aligned}$$

As in previous sections, Bayesian estimation can be performed using noninformative priors. Regression coefficients can follow diffuse normal priors (e.g., $N(0,10^{6})$), while the residual variance can be assigned a Gamma prior (e.g., *Gamma*(0.001, 0.001)).

#### With ML estimation

Although R packages ’censReg’ and ’survival’ can be used for modeling censored data in a single outcome variable, they do not accommodate censored data in predictors or moderators. While the censored data specification in Mplus allows for modeling ceiling or floor effects in a single observed variable (e.g., an outcome or a predictor), it does not support such a specification for the interaction term. Our pilot simulation studies showed nonignorable biases, inflated Type I errors, and under-coverage for estimating and testing $\beta _{3}$ when applying the censored data specification in Mplus to the observed variables (e.g., the outcome, focal predictor, and moderator variables) without additional treatments to the interaction term (see Table S18 in the online Supplemental Materials).

To address these limitations, we propose integrating Tobit modeling with latent variable modeling to handle ceiling or floor effects for moderated regression. We used Mplus to implement the approach and utilized its existing functions for censored data and latent variable modeling. The moderated regression model can be specified in the latent variable modeling framework as:

19 $$\begin{aligned} \begin{array}{c} \eta _{i}=\beta _{0}+\left( \begin{array}{ccc} \beta _{1} & \beta _{2} & \beta _{3}\end{array}\right) \left( \begin{array}{c} \xi _{1i}\\ \xi _{2i}\\ \xi _{1i}\times \xi _{2i} \end{array}\right) +\zeta _{i}\\ x_{i}^{*}=0+1\times \xi _{1i}+\delta _{1i}\\ z_{i}^{*}=0+1\times \xi _{2_{i}}+\delta _{2i}\\ y_{i}^{*}=0+1\times \eta _{i}+\epsilon _{i} \end{array}, \end{aligned}$$

where $y^{*}$, $x^{*}$, and $z^{*}$ are the true scores after using the censored data specifications in Mplus, and are modeled as outcomes in the measurement models. Specifically, $\eta$ represents a latent endogenous variable modeling the outcome $Y^{*}$, while $\xi _{1}$ and $\xi _{2}$ are latent exogenous variables modeling the focal predictor $X^{*}$ and the moderator $Z^{*}$, respectively. The interaction term $\xi _{1}\times \xi _{2}$ is also treated as a latent variable that is formed by the product of the two latent exogenous variables.

The path coefficients $\beta$s in Eq. 19 are mathematically equivalent to the regression coefficients in Eq. 18. $\zeta _{i}$ in Eq. 19 is the error term of the structural model and is equivalent to $e_{i}$ in the regression format in Eq. 18. $\zeta _{}$ is uncorrelated with the errors ($\delta _{1}$, $\delta _{2}$, and $\epsilon _{3}$) in the measurement models. In each measurement model, only a single indicator is included for each latent variable. For model identification, each measurement model is set to have an intercept of 0, a factor loading of 1, and a very small error variance (e.g., $var(\delta _{1})=var(\delta _{2})=var(\epsilon )=.01$). Under these constraints, the mean of each latent variable is equal to the mean of the corresponding true scores (e.g., $mean(\xi _{1})=mean(x^{*})$) and the variance of each latent variable closely approximate the variance of the corresponding true scores (e.g., $var(\xi _{1})=var(x^{*})-.01$). With this setup, the ceiling or floor effects in the interaction term are properly treated when the focal predictor and/or the moderator are subject to these effects.

The likelihood function of the observed scores $y_{i}$, $x_{i}$, and $z_{i}$ is complex due to the many possible scenarios. Given that each of these three variables can take one of three possible values (free of floor or ceiling effects, at the floor, or at the ceiling), there are in total 27 possible cases. To simplify the presentation, we illustrate the likelihood function for a specific example, where the outcome and focal predictor variables are subject to ceiling effects with ceiling thresholds of $b_{Y}$ and $b_{X}$, respectively, and the moderator is free of ceiling or floor effects. In this case, there are four possible likelihood expressions:

20 $$\begin{aligned} l_{i}={\left\{ \begin{array}{ll} f(x_{i},z_{i},y_{i}|\Theta ) & \textrm{if}\,x_{i}<b_{X},y_{i}<b_{Y}\\ \intop _{b_{Y}}^{\infty }f(x_{i},z_{i},y_{i}|\Theta )dY_{i} & \textrm{if}\,x_{i}<b_{X},y_{i}=b_{Y}\\ \intop _{b_{X}}^{\infty }f(x_{i},z_{i},y_{i}|\Theta )dX_{i} & \textrm{if}\,x_{i}=b_{X},y_{i}<b_{Y}\\ \intop _{b_{Y}}^{\infty }\intop _{b_{X}}^{\infty }f(x_{i},z_{i},y_{i}|\Theta )dX_{i}dY_{i} & \textrm{if}\,x_{i}=b_{X},y_{i}=b_{Y} \end{array}\right. }, \end{aligned}$$

where $\Theta$ contains all model parameters and latent variables, and *f* is the joint distribution of $x_{i}$, $z_{i}$, and $y_{i}$. The full likelihood function of the model is:

21 $$\begin{aligned} L=\prod _{i}\int l_{i}N(\mathbf {\boldsymbol{\pi }}_{i};\boldsymbol{\mathbf {\mu }_{\pi }},\boldsymbol{D})d\mathbf {\boldsymbol{\pi }}_{i}, \end{aligned}$$

where $\mathbf {\boldsymbol{\pi }}_{i}=(\eta _{1i},\xi _{1i},\xi _{2i})$ and $\mathbf {\boldsymbol{\pi }}_{i}\sim N(\mathbf {\mathbf {\mu }}_{\pi },\boldsymbol{D})$ with $\mathbf {\mathbf {\mu }}_{\pi }$ representing the means of the latent variables and $\boldsymbol{D}$ denoting the covariance matrix of the latent variables.

Despite the added complexity, integrating Tobit moderated regression with the latent variable modeling framework offers potential advantages. This approach allows for seamless extensions to multivariate models with multiple outcomes and multiple predictors/moderators (e.g., path analysis) to address ceiling or floor effects. Additionally, it enables the implementation of ML estimation in widely used latent variable modeling software such as Mplus, facilitating easy extensions to research settings with ceiling or floor effects where the analysis model of interest is a latent variable model.

#### A simulation study

We conducted a simulation study to evaluate the performance of the three approaches for addressing ceiling/floor effects in moderated regression analysis: the conventional approach, the proposed Tobit approach with ML estimation (Tobit ML), and the proposed Tobit approach with Bayesian estimation (Tobit Bayesian). Similar to the simulation study in the dependent-sample *t*-test section, we focused on the scenario of having ceiling data as an example and the findings can be generalized to scenarios with floor data when the true population distribution is symmetrical.

#### Simulation design

Two scenarios were considered: (1) the focal predictor and outcome variables have ceiling effects or (2) the moderator has ceiling effects. Motivated by the real data analysis example presented later in this section, for Scenario 1, we considered the following setting: the focal predictor was the pre-test variable and the outcome was the post-test so the two variables shared the same ceiling threshold, and the ceiling proportion of focal predictor *X* was manipulated with $CP=$ 0%, 10%, 20%, and 30% while the moderator had no ceiling effects. For Scenario 2, the ceiling proportion of the moderator was manipulated with $CP=$ 0%, 10%, 20%, and 30% while the focal predictor and outcome variables had no ceiling effects. The regression intercept was set to 0 ($\beta _{0}=0$), the regression coefficients of the main effects were set to 1 ($\beta _{1}=\beta _{2}=1$), and the variance of prediction error *e* was set to 1 across all conditions. The regression coefficient of the interaction term $\beta _{3}$ was set to be 0 and .39 for null and medium effect size (corresponding to $f^{2}$ of 0 and around 0.15, respectively). In addition, the following two factors were manipulated: the correlation between the focal predictor variable and the moderator variable ($\rho =0$ or $\rho =0.3$, corresponding to null or medium levels) and the sample size ($N=$ 50, 100, 200, and 500). A total of $2\times 4\times 2\times 2\times 4=128$ simulation conditions were considered and each condition had 1000 replications.

True scores of the focal predictor and moderator were generated from a bivariate normal distribution with marginal means of 0s, marginal variances of 1, and the correlation between the focal predictor and moderator in each specific condition. True scores of the outcome variable were generated through the moderated regression model in Eq. 18, with the specified regression coefficients in the condition and normally distributed regression residuals. Then, with the ceiling proportions in the condition, certain proportions of the focal predictor, moderator, and outcome variable scores were set to be their ceiling thresholds. In each replication of the Bayesian analysis, two Markov chains were used, each with a total of a burn-in period of 30,000 iterations and 100,000 post-burn-in iterations. The performance of the three modeling approaches was also evaluated based on the estimation accuracy and coverage of the regression coefficients (main effects: $\beta _{1}$ and $\beta _{2}$; interaction effect: $\beta _{3}$) using estimates from converged replications. Convergence, estimation accuracy, and coverage were assessed using the same criteria as those used for the dependent-sample *t*-test simulation study.

**Table 3:** Moderated regression simulation results: Empirical bias and relative bias of the regression coefficient $\beta _{3}$ from Scenario 1 where the outcome and the focal predictor have ceiling effects

	CP = 0%	CP = 10%			CP = 20%			CP = 30%		
			Tobit	Tobit		Tobit	Tobit		Tobit	Tobit
N	Reference	Conventional	ML	Bayesian	Conventional	ML	Bayesian	Conventional	ML	Bayesian
Empirical bias under $\beta _{3}=0$ and $\rho =0.3$										
50	-.003	**-.256**	.010	.047	**-.280**	.032	.078	**-.317**	.037	**.103**
100	.004	**-.238**	.009	.028	**-.287**	.002	.031	**-.316**	.006	.046
200	.001	**-.243**	.001	.012	**-.282**	.003	.022	**-.311**	-.002	.025
500	-.001	**-.239**	.002	.009	**-.277**	.001	.014	**-.309**	-.001	.019
Relative bias under $\beta _{3}=0.39$ and $\rho =0.3$										
50	-.004	**-.924**	.079	**.167**	**-1.008**	.053	**.170**	**-1.050**	.057	**.200**
100	-.011	**-.927**	.035	.072	**-.991**	.018	.069	**-1.012**	.012	.089
200	.002	**-.929**	.026	.040	**-.997**	-.008	.019	**-.998**	.004	.052
500	.000	**-.921**	.013	.015	**-.977**	.011	.021	**-.982**	.002	.027
Empirical bias under $\beta _{3}=0$ and $\rho =0$										
50	.002	**-.185**	.009	.028	**-.240**	.007	.027	**-.284**	.015	.039
100	-.002	**-.190**	-.003	.005	**-.232**	.005	.015	**-.273**	.003	.014
200	-.003	**-.184**	.001	.006	**-.226**	.006	.012	**-.268**	.001	.006
500	.000	**-.183**	-.002	.000	**-.229**	-.003	.000	**-.268**	-.003	.000
Relative bias under $\beta _{3}=0.39$ and $\rho =0$										
50	.007	**-.696**	.038	.084	**-.813**	-.011	.052	**-.861**	.023	**.101**
100	-.012	**-.704**	.019	.035	**-.792**	.025	.048	**-.831**	.017	.052
200	.008	**-.682**	.015	.019	**-.769**	.016	.024	**-.818**	.007	.023
500	-.002	**-.679**	.016	.012	**-.766**	.003	.003	**-.813**	.009	.012

Notes. CP: ceiling proportion; $\rho$: the correlation between the focal predictor and the moderator. Empirical bias values greater than .10 and relative bias values greater than 10% are bold

### Results

We reported the primary results from Scenario 1 where the outcome and the focal predictor had ceiling effects in the main text. Tables 3 and 4 display the simulation results for the regression coefficient of the interaction effect $\beta _{3}$ from Scenario 1, as this parameter is of particular research interest. Findings for the main effects ($\beta _{1}$ and $\beta _{2}$) from Scenario 1 were similar to those for the interaction effect $\beta _{3}$ and are presented in the online supplemental materials (Tables S7 to S10). Additionally, findings from Scenario 2 where the moderator had ceiling effects, were similar to those from Scenario 1 and the results are also reported in the online supplemental materials (Tables S11 to S17).

Both the conventional and Tobit Bayesian approaches had 100% convergence rate (see Tables S6 and S11 in the online supplements). However, the Tobit ML approach showed lower convergence in certain conditions (e.g., when *N* = 50 or 100 with *CP* = 30% in Scenario 1), with convergence rates as low as 81.8% (see Table S6 in the online supplements).

The conventional approach performed poorly in estimating coefficients $\beta _{3}$, leading to substantial relative biases (see Table 3). For example, under the condition of $\beta _{3}=0.39$, $\rho =0.3$, and $N=100$, the relative biases of $\beta _{3}$ were 92.7%, 99.1%, and 101.2% when *CP* = 10%, 20%, and 30%, respectively. Unsatisfactory estimation accuracy was also observed for coefficients $\beta _{1}$ and $\beta _{2}$ (see Tables S7 and S8 in the online supplements). The biases were larger when the ceiling proportion or the correlation between the focal predictor and moderator was greater. Additionally, the conventional approach had inflated empirical Type I error rates and severe under-coverage problems (see Table 4). For example, when $\rho =0.3$ and $N=100$, the empirical Type I error rates of $\beta _{3}$ were 69.8%, 80.3%, and 79.7% and the coverage rates of $\beta _{3}$ were 7.9%, 7.3%, and 8.0% when *CP* = 10%, 20%, or 30%, respectively. The empirical Type I error rates and the coverage rates were slightly better when *CP* = 30% compared to *CP* = 20%, although all showed poor performance of the conventional approach. This pattern of results was due to both the higher biases and wider confidence intervals (more uncertainty) in conditions with *CP* = 30% compared to those with *CP* = 20%. Unsatisfactory inference results were also observed for coefficients $\beta _{1}$ and $\beta _{2}$ (see Tables S9 and S10 in the online supplements).

The proposed Tobit modeling approaches, using either ML or Bayesian estimation, outperformed the conventional approach in both estimation accuracy and coverage across all studied conditions. Furthermore, both Tobit approaches demonstrated satisfactory estimation accuracy, empirical Type I error rates, and coverage rates in almost all studied conditions. There were a few exceptions. The Tobit Bayesian approach had elevated bias for $\beta _{3}$ when the sample size was 50 (see Table 3), and the Tobit ML approach had slightly elevated empirical Type I error rates (7.6% to 8.6%) in three conditions where the sample size was small (50 or 100) with higher CP (see Tables 4 and S17).

**Table 4:** Moderated regression simulation results: Empirical Type I error rates and coverage rates (%) of the regression coefficient $\beta _{3}$ from Scenario 1 where the outcome and the focal predictor have ceiling effects

	CP = 0%	CP = 10%			CP = 20%			CP = 30%		
			Tobit	Tobit		Tobit	Tobit		Tobit	Tobit
N	Reference	Conventional	ML	Bayesian	Conventional	ML	Bayesian	Conventional	ML	Bayesian
Empirical Type I error rates under $\beta _{3}=0$ and $\rho =0.3$										
50	6.4	**49.2**	5.7	5.1	**52.4**	7.0	5.6	**54.5**	**8.6**	7.0
100	5.3	**69.8**	6.0	5.2	**80.3**	6.1	5.3	**79.7**	6.7	5.2
200	5.4	**94.4**	5.9	5.3	**97.5**	5.3	6.2	**97.6**	5.1	4.3
500	4.6	**100.0**	4.7	5.2	**100.0**	6.8	6.6	**100.0**	4.9	4.8
Coverage rates under $\beta _{3}=0.39$ and $\rho =0.3$										
50	93.6	**31.1**	93.3	93.8	**30.0**	94.1	95.9	**29.7**	91.9	93.9
100	93.8	**7.9**	94.0	94.6	**7.3**	93.3	94.0	**8.0**	94.9	96.3
200	94.8	**0.3**	93.2	93.7	**0.1**	93.5	93.9	**0.6**	95.6	95.5
500	94.4	**0.0**	95.9	96.0	**0.0**	94.9	95.4	**0.0**	95.6	96.2
Empirical Type I error rates under $\beta _{3}=0$ and $\rho =0$										
50	6.2	**29.0**	5.9	5.0	**39.6**	6.7	6.1	**47.5**	7.2	5.0
100	5.3	**51.7**	6.0	5.0	**62.5**	**7.6**	5.8	**67.6**	6.0	5.2
200	4.5	**75.3**	4.8	4.5	**88.1**	4.7	4.0	**91.3**	6.6	5.6
500	5.1	**98.4**	4.7	4.8	**99.8**	7.2	7.1	**100.0**	4.4	4.6
Coverage rates under $\beta _{3}=0.39$ and $\rho =0$										
50	93.0	**51.0**	94.5	96.1	**42.9**	92.8	94.9	**45.3**	94.1	95.9
100	94.6	**24.1**	92.7	94.0	**20.9**	94.3	95.2	**23.5**	95.0	95.7
200	95.7	**5.5**	94.3	94.8	**3.9**	94.0	93.7	**3.5**	93.4	93.9
500	95.6	**0.0**	95.7	95.8	**0.0**	94.5	94.6	**0.0**	94.2	93.9

Notes. CP: ceiling proportion; $\rho$: the correlation between the focal predictor and the moderator. Empirical Type I error rates outside the 2.5–7.5% range and coverage rates outside the 91–98% range were highlighted in bold

**Table 5:** Moderated regression real data analysis results

Parameter	Conventional	Tobit ML	Tobit Bayesian
$\beta _{0}$	-5.10 (-13.33, 3.13)	na	-3.63 (-16.60, 7.97)
$\beta _{1}$	1.37 (0.59, 2.16)	na	1.21 (0.11, 2.49)
$\beta _{2}$	1.98 (0.28, 3.68)	na	1.45 (-0.96, 4.03)
$\beta _{3}$	-0.16 (-0.32, 0.00)	na	-0.10 (-0.35, 0.12)

Note. na: not available. Point estimates are outside the parentheses. 95% confidence or credible intervals are in the parentheses

### An illustrative real data analysis example

We used the same real data in the dependent-sample *t*-test example to illustrate the applications of the modeling approaches for moderated regression. The conventional moderated regression model is

22 $$\begin{aligned} y_{2i}=\beta _{0}+\beta _{1}y_{1i}+\beta _{2}z_{i}+\beta _{3}y_{1i}z_{i}+e_{i} \end{aligned}$$

$Y_{1}$ and $Y_{2}$ contain the observed recall test scores from the two repeated measurements; 33.3% and 41.7% of $Y_{1}$ and $Y_{2}$ were ceiling data and thus ceiling effects were substantial in the focal predictor $Y_{1}$ and outcome $Y_{2}$. Moderator *Z* contains the Wechsler Adult Intelligence Scale (WAIS) III vocabulary scores and does not have ceiling effects.

The Tobit model with ML estimation did not converge. The nonconvergence was not surprising, as we found in the simulation study that the convergence rate was 85.3% with $N=100$, *CP* = 30%, and $\beta _{3}$=0 when both the outcome and the focal predictor had ceiling effects. In contrast, the conventional and Tobit Bayesian approaches achieved convergence, and their results are displayed in Table 5. The moderation effect $\beta _{3}$ was not statistically significant from either approach. However, the main effect of the moderator $\beta _{2}$ was statistically significant from the conventional approach but was not from the Tobit Bayesian approach. Thus, there were different statistical findings from the two approaches. The simulation results showed that the conventional approach can yield inflated Type I error rates for the regression coefficients when ceiling effects exist. Thus, we suggest the use of the Tobit Bayesian approach rather than the conventional approach for this example to address ceiling effects.

To facilitate the implementation of the Tobit modeling approaches with ML and Bayesian estimation for moderated regression analysis, we shared a simulated data set from the simulation condition under Scenario 1 with focal predictor $CP=30\%$, $\beta _{3}=0$, $\rho =0.3$, and $N=100$ as well as the Mplus and R JAGS scripts in the online Supplemental Materials.

## Discussion

Ceiling or floor effects are common in behavioral research. Although statistical approaches have been developed to address these effects for a variety of statistical tests or models, there is limited research on developing and evaluating statistical approaches to handle these effects for widely used statistical tests and models such as the dependent-sample *t*-test and moderated regression. In this study, we introduced novel Tobit modeling approaches to account for ceiling and floor effects in repeated measures, outcome, and predictor/moderator variables. The proposed approaches were estimated using maximum likelihood (ML) or Bayesian estimation and implemented in established software programs (Mplus and R JAGS). Our simulation results showed that the proposed approaches with ML or Bayesian estimation performed well in handling ceiling or floor effects for the dependent-sample *t*-test and moderated regression. In contrast, the conventional approach where ceiling or floor data were treated as if true values led to biased estimates and inaccurate inference conclusions, with even as low as 10% ceiling data. The real data analysis examples further demonstrated that misleading conclusions can be made from the conventional approach.

Originally developed to address limited or censored data in the dependent variable for regression analysis (Tobin, 1958), Tobit modeling has been extended to handle ceiling/floor effects in outcome variables for independent-sample *t* tests and ANOVA (Liu & Wang, 2021) and address these effects in outcome and mediator variables for mediation analysis (Wang & Zhang, 2011). It has also been integrated with latent variable modeling to handle censored data for confirmatory factor analysis (Muthen, 1989) and ceiling effects for latent growth curve modeling (Wang et al., 2008). In this extended research, the observed variables with censored or ceiling data were modeled as observed dependent or exogenous variables in the focal models of interest, making the application of Tobit modeling straightforward. However, in the dependent-sample *t*-test with ceiling or floor effects in both samples (e.g., pre-test and post-test), it is challenging to model the covariance between the two censored variables. In moderated regression, the variables exhibiting ceiling or floor effects can be the observed predictor or moderator variables. In this case, the interaction term is also subject to ceiling or floor effects, making the application of Tobit modeling challenging, especially when ML is used for estimation. To overcome the challenges, we integrated Tobit modeling with latent variable modeling to handle ceiling or floor data for the dependent-sample *t*-test and moderated regression, even though the analysis models of interest did not involve latent variables. Specifically, the observed variables with ceiling or floor data were modeled with censored normal distributions; and the true scores after addressing ceiling or floor effects were modeled as outcomes in measurement models. The latent variables in the measurement models were then modeled in a structural model to estimate parameters in the analysis models of interest. Note that the measurement models and the structural model are estimated simultaneously. This integrated approach can be readily extended to other models, such as path analysis or structural equation models, to address ceiling or floor effects in predictors, mediators, moderators, and outcomes.

To address ceiling or floor effects in the dependent-sample *t*-test and moderated regression, the proposed Tobit model specifications under Bayesian estimation are relatively more intuitive and straightforward than those under ML estimation. However, some researchers may be more familiar with ML than Bayesian methods. Thus, we provided both estimation methods for the proposed Tobit modeling approaches. In prior research of Tobit modeling, one estimation method (ML or Bayesian) was used and evaluated at a time. In this study, we evaluated the performance of both estimation methods. For the dependent-sample *t*-test, both estimation methods worked well for estimating the proposed Tobit models across all studied simulation conditions (e.g., the sample size can be as small as 50 and the ceiling proportion can be as high as 30%). In contrast, the conventional approach had satisfactory results only when the mean difference is zero and the population variances of pre-test and post-test scores are equal, where the ceiling effects are canceled out in the pre-test and post-test comparisons. In practice, researchers do not know whether the true mean difference is zero and whether the two population variances are equal. The former actually is the research question to be answered. When the true mean difference is not zero and/or when the two population variances are not equal, our simulation results showed that the conventional approach yielded poor performance. Therefore, we recommend the use of our proposed Tobit modeling approaches with ML or Bayesian estimation over the conventional approach to handle ceiling or floor effects for the dependent-sample *t*-test.

For moderated regression, both estimation methods produced satisfactory results when the sample size was not small (e.g., 200 or more). However, under certain conditions when the sample size was small (e.g., 50 or 100) and/or the ceiling proportion was relatively large (e.g., 30%), ML estimation implemented in Mplus exhibited convergence issues (with nonconvergence rates as high as 19%) and slightly inflated Type I error rates (up to 8.6%). Additionally, Bayesian estimation implemented in R JAGS showed elevated biases in regression coefficient estimates (up to 20%) when the sample size was very small (e.g., 50). Despite these limitations in extreme conditions, both ML and Bayesian approaches consistently outperformed the conventional approach that ignores ceiling or floor effects. Therefore, we also recommend the use of our proposed Tobit modeling approaches with ML or Bayesian estimation to handle ceiling or floor effects for moderated regression. When nonconvergence occurs from Tobit modeling with ML estimation for moderated regression, Tobit modeling with Bayesian estimation could be used as long as the sample size is not too small (e.g., 50).

It is important to acknowledge the assumptions in our proposed approaches. First, we assumed that the true scores, after adjusting for ceiling or floor effects, followed a normal distribution. This assumption was fundamental to the construction of the likelihood function, which was based on the standard normal density and cumulative normal density functions. If the normality assumption is violated, estimation and inference accuracy may be compromised (Cain et al., 2017), warranting future research into the robustness of the proposed approaches under non-normal true scores. Second, we assumed the absence of missing data in variables affected by ceiling or floor effects in the current study. However, ceiling/floor effects and missingness can co-occur in the same variable. For a variable, it could occur that some participants have ceiling effects, some participants have missingness, whereas the true scores are observed for the rest of the participants. Although full information ML and Bayesian methods can handle missing data under missing completely at random (MCAR) and missing at random (MAR) mechanisms (e.g., Little & Rubin, 2002; Zhang and Wang, 2017), the performance of our proposed approaches in the presence of missing data remains an open question. Thus, how well the proposed approaches perform under the presence of missing data should be studied in future research. Third, measurement errors (McDonald, 1999) were not explicitly modeled in the variables with ceiling or floor effects. While our Tobit modeling approach employs latent variable modeling, each latent variable has only one manifest indicator, forming a single-indicator model. When measurement errors exist, extending the proposed approaches to handle both ceiling/floor effects and measurement errors is an interesting future research direction.

In summary, this study proposed and evaluated novel Tobit modeling approaches using ML or Bayesian estimation to address ceiling and floor effects in the dependent-sample *t*-test and moderated regression. Our simulation results demonstrated that the proposed approaches outperformed the conventional approach across all studied simulation conditions and performed satisfactorily under most simulation conditions. We hope that these developments will provide researchers with effective tools for handling ceiling and floor effects in their research.

## Supplementary Information

Below is the link to the electronic supplementary material.

Supplementary file 1 (pdf 480 KB)

## Data Availability

Illustrative data sets as well as Mplus and R JAGS scripts are available in the online supplemental materials (https://github.com/peggywangnd/Tobitmodelexamples).

## Footnotes

1 Although it is not common to have zero covariance or correlation between pre-test and post-test true scores, we included the zero-correlation level to test the robustness of the proposed Tobit modeling approaches, as this covariance is freely estimated in the proposed approaches regardless of whether its true value is zero or not.
