# Supplementary material for: Tobit modeling for dependent-sample t-tests and moderated regression with ceiling or floor data
Source: Behav Res Methods. 2025 Dec 10;58(1):23. doi: 10.3758/s13428-025-02904-y (PMC12695936; doi:10.3758/s13428-025-02904-y)
Supplement: Supplementary file 1 — (pdf 480 KB) [file 13428_2025_2904_MOESM1_ESM.pdf]

Online supplemental materials for "Tobit modeling for dependent-sample t-tests and moderated regression with ceiling or floor data"

Table S1. The dependent-sample t-test simulation results: Convergence rates (%) for conditions with $\rho = 0.5$

		CP = 0%	CP = 10%			CP = 20%			CP = 30%		
		Reference	Conventional	Tobit ML	Tobit Bayesian	Conventional	Tobit ML	Tobit Bayesian	Conventional	Tobit ML	Tobit Bayesian
		Convergence rates under Cohen's $d = 0$									
N = 50	*SDR = 1*	100	100	100	100	100	100	100	100	100	100
	SDR = 1.5	100	100	100	100	100	100	100	100	100	100
N = 100	*SDR = 1*	100	100	100	100	100	100	100	100	100	100
	SDR = 1.5	100	100	100	100	100	100	100	100	100	100
N = 200	*SDR = 1*	100	100	100	100	100	100	100	100	100	100
	SDR = 1.5	100	100	100	100	100	100	100	100	100	100
N = 500	*SDR = 1*	100	100	100	100	100	100	100	100	100	100
	SDR = 1.5	100	100	100	100	100	100	100	100	100	100
		Convergence rates under Cohen's $d = 0.5$									
N = 50	*SDR = 1*	100	100	100	100	100	100	100	100	100	100
	SDR = 1.5	100	100	100	100	100	100	100	100	100	100
N = 100	*SDR = 1*	100	100	100	100	100	100	100	100	100	100
	SDR = 1.5	100	100	100	100	100	100	100	100	100	100
N = 200	*SDR = 1*	100	100	100	100	100	100	100	100	100	100
	SDR = 1.5	100	100	100	100	100	100	100	100	100	100
N = 500	*SDR = 1*	100	100	100	100	100	100	100	100	100	100
	SDR = 1.5	100	100	100	100	100	100	100	100	100	100

Note. CP: ceiling proportion of pretest scores; SDR: the population standard deviation ratio.

Table S2. The dependent-sample t-test simulation results: Convergence rates (%) for conditions with $\rho = 0$

		CP = 0%	CP = 10%			CP = 20%			CP = 30%		
		Reference	Conventional	Tobit ML	Tobit Bayesian	Conventional	Tobit ML	Tobit Bayesian	Conventional	Tobit ML	Tobit Bayesian
		Convergence rates under Cohen's $d = 0$									
N = 50	*SDR = 1*	100	100	100	100	100	100	100	100	100	100
	SDR = 1.5	100	100	100	100	100	100	100	100	100	100
N = 100	*SDR = 1*	100	100	100	100	100	100	100	100	100	100
	SDR = 1.5	100	100	100	100	100	100	100	100	100	100
N = 200	*SDR = 1*	100	100	100	100	100	100	100	100	100	100
	SDR = 1.5	100	100	100	100	100	100	100	100	100	100
N = 500	*SDR = 1*	100	100	100	100	100	100	100	100	100	100
	SDR = 1.5	100	100	100	100	100	100	100	100	100	100
		Convergence rates under Cohen's $d = 0.5$									
N = 50	*SDR = 1*	100	100	100	100	100	100	100	100	100	100
	SDR = 1.5	100	100	100	100	100	100	100	100	100	100
N = 100	*SDR = 1*	100	100	100	100	100	100	100	100	100	100
	SDR = 1.5	100	100	100	100	100	100	100	100	100	100
N = 200	*SDR = 1*	100	100	100	100	100	100	99.9	100	100	100
	SDR = 1.5	100	100	100	100	100	100	100	100	100	100
N = 500	*SDR = 1*	100	100	100	100	100	100	100	100	100	100
	SDR = 1.5	100	100	100	100	100	100	100	100	100	100

Note. CP: ceiling proportion of pretest scores; SDR: the population standard deviation ratio.

Table S3. The dependent-sample t-test simulation results: Empirical bias and relative bias of mean difference estimates from conditions with $\rho = 0$

		CP = 0%	CP = 10%			CP = 20%			CP = 30%		
		Reference	Conventional	Tobit ML	Tobit Bayesian	Conventional	Tobit ML	Tobit Bayesian	Conventional	Tobit ML	Tobit Bayesian
		Estimation empirical bias under Cohen's $d = 0$									
N = 50	*SDR = 1*	0.006	-0.008	-0.009	-0.004	-0.003	-0.003	0.004	0.002	0.002	0.014
	SDR = 1.5	-0.008	-0.102	0.017	0.007	-0.154	0.006	-0.005	-0.184	-0.007	-0.018
N = 100	*SDR = 1*	0.007	-0.001	-0.001	0.001	-0.005	-0.006	0.000	0.002	0.005	0.012
	SDR = 1.5	0.001	-0.112	0.007	0.002	-0.164	-0.004	-0.008	-0.182	0.005	-0.007
N = 200	*SDR = 1*	-0.001	0.004	0.004	0.005	0.001	0.001	0.005	0.000	-0.002	0.005
	SDR = 1.5	-0.006	-0.117	0.000	0.000	-0.162	-0.003	-0.003	-0.179	0.004	-0.002
N = 500	*SDR = 1*	0.001	-0.003	-0.004	-0.001	0.000	0.000	0.002	0.000	-0.001	0.004
	SDR = 1.5	-0.001	-0.117	-0.001	0.000	-0.156	0.003	0.001	-0.180	0.003	0.000
		Estimation relative bias under Cohen's $d = 0.5$									
N = 50	*SDR = 1*	0.003	**-0.190**	-0.007	0.030	**-0.303**	0.005	0.070	**-0.433**	0.008	0.084
	SDR = 1.5	-0.016	**-0.424**	0.009	0.024	**-0.569**	0.013	0.022	**-0.684**	0.013	0.010
N = 100	*SDR = 1*	0.002	**-0.184**	-0.002	0.024	**-0.311**	0.010	0.051	**-0.439**	-0.004	0.048
	SDR = 1.5	0.004	**-0.423**	0.002	0.014	**-0.576**	-0.006	-0.005	**-0.683**	0.001	-0.007
N = 200	*SDR = 1*	-0.003	**-0.180**	0.004	0.023	**-0.310**	0.009	0.039	**-0.435**	-0.002	0.040
	SDR = 1.5	-0.007	**-0.422**	0.001	0.013	**-0.575**	0.000	0.005	**-0.682**	0.010	0.008
N = 500	*SDR = 1*	0.004	**-0.183**	-0.001	0.012	**-0.315**	0.002	0.022	**-0.435**	-0.001	0.024
	SDR = 1.5	0.002	**-0.422**	0.002	0.011	**-0.573**	0.002	0.009	**-0.683**	0.004	0.009

Note. CP: ceiling proportion; SDR: the population standard deviation ratio. Unsatisfactory results are highlighted in bold.

Table S4. The dependent-sample t-test simulation results: Empirical Type I error rates (%) and coverage rates (%) of mean difference estimates from conditions with $\rho = 0$

		CP = 0%	CP = 10%			CP = 20%			CP = 30%		
		Reference	Conventional	Tobit ML	Tobit Bayesian	Conventional	Tobit ML	Tobit Bayesian	Conventional	Tobit ML	Tobit Bayesian
		Empirical Type I error rates (%) under Cohen's $d = 0$									
N = 50	*SDR = 1*	5.1	3.6	4.2	4.5	4.9	6.0	6.0	4.7	6.0	5.8
	SDR = 1.5	5.2	6.9	6.7	6.5	**11.1**	5.3	4.8	**16.3**	6.0	6.4
N = 100	*SDR = 1*	5.5	4.9	5.0	4.8	5.3	5.2	5.4	4.5	5.1	5.2
	SDR = 1.5	3.7	**10.8**	5.7	6.1	**20.3**	4.5	4.5	**25.1**	4.7	5.4
N = 200	*SDR = 1*	4.9	5.5	5.4	5.5	4.9	5.7	5.6	5.1	4.9	5.8
	SDR = 1.5	4.9	**18.2**	5.1	5.5	**36.7**	4.6	5.2	**49.7**	6.2	6.3
N = 500	*SDR = 1*	5.5	5.6	5.7	6.5	3.7	4.3	4.4	5.5	5.1	5.9
	SDR = 1.5	5.5	**38.8**	5.7	6.1	**70.0**	5.1	5.2	**88.0**	5.9	6.4
		Coverage rates (%) under Cohen's $d = 0.5$									
N = 50	*SDR = 1*	94.6	**88.3**	93.9	94.1	**70.0**	96.1	94.3	**37.0**	95.1	93.2
	SDR = 1.5	94.8	**49.7**	95.4	94.4	**13.6**	94.6	94.0	**1.4**	94.6	93.4
N = 100	*SDR = 1*	95.9	**82.5**	93.8	94.6	**44.2**	95.5	94.9	**8.5**	95.8	94.2
	SDR = 1.5	95.0	**20.3**	93.8	93.6	**0.7**	96.1	95.2	**0.0**	96.0	95.2
N = 200	*SDR = 1*	94.0	**67.7**	95.4	95.1	**16.1**	94.5	94.7	**0.3**	95.4	94.5
	SDR = 1.5	94.7	**1.8**	94.9	95.3	**0.0**	94.8	94.5	**0.0**	96.0	95.9
N = 500	*SDR = 1*	94.3	**32.7**	94.8	94.5	**0.5**	95.4	95.4	**0.0**	95.1	95.1
	SDR = 1.5	95.5	**0.0**	95.5	95.9	**0.0**	96.3	94.9	**0.0**	95.1	95.2

Note. CP: ceiling proportion; SDR: the population standard deviation ratio. Unsatisfactory results are highlighted in bold.

Table S5. The dependent-sample t-test simulation results: Empirical Type I error rates (%) and coverage rates (%) of mean difference estimates when using the t-distributions with the degrees of freedom N- 1 to construct confidence intervals for the Tobit ML approach

		$\rho = 0$			$\rho = 0.5$		
CP of pre-test scores =		10%	20%	30%	10%	20%	30%
		Empirical Type I error rates (%) under Cohen's $d = 0$					
N = 50	*SDR = 1*	3.5	5.2	5.1	4.4	4.7	4.6
	SDR = 1.5	6.2	5.0	5.2	4.5	5.1	4.4
N = 100	*SDR = 1*	4.2	5.0	5.0	3.7	5.4	5.1
	SDR = 1.5	5.0	4.2	4.6	5.6	4.6	5.4
N = 200	*SDR = 1*	5.4	5.6	4.7	4.7	4.9	4.9
	SDR = 1.5	4.8	4.4	5.6	4.8	3.7	4.6
N = 500	*SDR = 1*	5.7	4.3	5.1	5.4	4.0	4.5
	SDR = 1.5	5.7	5.1	5.9	4.4	4.8	5.6
		Coverage rates (%) under Cohen's $d = 0.5$					
N = 50	*SDR = 1*	94.7	96.6	95.4	95.1	95.5	95.4
	SDR = 1.5	96.2	95.5	94.9	94.5	95.5	95.8
N = 100	*SDR = 1*	93.8	95.9	96.0	96.6	95.3	94.9
	SDR = 1.5	94.1	96.4	96.3	94.6	95.4	94.7
N = 200	*SDR = 1*	95.5	94.6	95.6	94.8	95.1	95.0
	SDR = 1.5	94.9	94.8	96.2	95.4	95.5	94.5
N = 500	*SDR = 1*	94.8	95.4	95.1	96.0	94.0	95.3
	SDR = 1.5	95.5	96.3	95.3	95.4	95.5	94.7

Note. CP: ceiling proportion; SDR: the population standard deviation ratio.

Table S6. Moderated regression simulation results: Convergence rates (%) for conditions in Scenario 1 where the outcome and the focal predictor have ceiling effects

		CP = 0%	CP = 10%			CP = 20%			CP = 30%		
		Reference	Conventional	Tobit ML	Tobit Bayesian	Conventional	Tobit ML	Tobit Bayesian	Conventional	Tobit ML	Tobit Bayesian
						$\rho = 0.3$					
N = 50	$\beta_3 = 0$	100.0	100.0	98.1	100.0	100.0	92.2	100.0	100.0	**81.8**	100.0
	$\beta_3 = 0.39$	100.0	100.0	99.3	100.0	100.0	93.7	100.0	100.0	**84.4**	100.0
N = 100	$\beta_3 = 0$	100.0	100.0	99.8	100.0	100.0	97.0	100.0	100.0	**85.3**	100.0
	$\beta_3 = 0.39$	100.0	100.0	99.9	100.0	100.0	96.4	100.0	100.0	**89.5**	100.0
N = 200	$\beta_3 = 0$	100.0	100.0	100.0	100.0	100.0	99.3	100.0	100.0	94.9	100.0
	$\beta_3 = 0.39$	100.0	100.0	100.0	100.0	100.0	99.4	100.0	100.0	94.4	100.0
N = 500	$\beta_3 = 0$	100.0	100.0	100.0	100.0	100.0	100.0	100.0	100.0	99.5	100.0
	$\beta_3 = 0.39$	100.0	100.0	100.0	100.0	100.0	100.0	100.0	100.0	99.2	100.0
						$\rho = 0$					
N = 50	$\beta_3 = 0$	100.0	100.0	99.0	100.0	100.0	94.2	100.0	100.0	90.2	100.0
	$\beta_3 = 0.39$	100.0	100.0	99.1	100.0	100.0	94.7	100.0	100.0	**86.2**	100.0
N = 100	$\beta_3 = 0$	100.0	100.0	99.9	100.0	100.0	99.0	100.0	100.0	93.6	100.0
	$\beta_3 = 0.39$	100.0	100.0	99.9	100.0	100.0	98.1	100.0	100.0	93.9	100.0
N = 200	$\beta_3 = 0$	100.0	100.0	100.0	100.0	100.0	99.4	100.0	100.0	98.2	100.0
	$\beta_3 = 0.39$	100.0	100.0	100.0	100.0	100.0	99.8	100.0	100.0	96.6	100.0
N = 500	$\beta_3 = 0$	100.0	100.0	100.0	100.0	100.0	100.0	100.0	100.0	100.0	100.0
	$\beta_3 = 0.39$	100.0	100.0	100.0	100.0	100.0	100.0	100.0	100.0	99.7	100.0

Note. CP: ceiling proportion. Unsatisfactory results are highlighted in bold.

Table S7. Moderated regression simulation results: Relative bias of regression coefficient β_1 for conditions in Scenario 1 where the outcome and the focal predictor have ceiling effects

		CP = 0%	CP = 10%			CP = 20%			CP = 30%		
		Reference	Conventional	Tobit ML	Tobit Bayesian	Conventional	Tobit ML	Tobit Bayesian	Conventional	Tobit ML	Tobit Bayesian
						$\rho = 0.3$					
N = 50	β_3= *0*	-0.007	**-0.164**	0.039	0.057	**-0.199**	0.027	0.056	**-0.203**	0.034	0.075
	β_3= *0.39*	-0.001	**-0.244**	0.029	0.047	**-0.276**	0.018	0.046	**-0.272**	0.037	0.068
N = 100	β_3= *0*	0.001	**-0.187**	0.004	0.005	**-0.197**	0.017	0.022	**-0.199**	0.015	0.030
	β_3= *0.39*	0.002	**-0.252**	0.021	0.023	**-0.280**	0.011	0.017	**-0.277**	0.006	0.021
N = 200	β_3= *0*	-0.002	**-0.180**	0.011	0.005	**-0.202**	0.013	0.009	**-0.201**	0.003	0.010
	β_3= *0.39*	0.002	**-0.253**	0.020	0.014	**-0.274**	0.009	0.005	**-0.275**	0.003	0.009
N = 500	β_3= *0*	-0.001	**-0.181**	0.012	0.002	**-0.200**	0.011	0.000	**-0.205**	-0.002	-0.005
	β_3= *0.39*	0.001	**-0.261**	0.010	-0.001	**-0.279**	0.009	-0.002	**-0.274**	0.004	0.001
						$\rho = 0$					
N = 50	β_3= *0*	0.003	**-0.153**	0.017	0.032	**-0.156**	0.028	0.057	**-0.160**	0.028	0.072
	β_3= *0.39*	0.002	**-0.201**	0.023	0.038	**-0.226**	0.014	0.039	**-0.236**	0.022	0.054
N = 100	β_3= *0*	-0.002	**-0.146**	0.015	0.017	**-0.160**	0.016	0.024	**-0.163**	0.010	0.028
	β_3= *0.39*	-0.003	**-0.210**	0.017	0.019	**-0.223**	0.021	0.029	**-0.227**	0.013	0.029
N = 200	β_3= *0*	0.002	**-0.151**	0.010	0.006	**-0.165**	0.008	0.008	**-0.162**	0.005	0.012
	β_3= *0.39*	0.000	**-0.215**	0.011	0.007	**-0.227**	0.013	0.013	**-0.230**	0.003	0.013
N = 500	β_3= *0*	0.000	**-0.151**	0.007	0.000	**-0.162**	0.008	0.003	**-0.160**	0.007	0.003
	β_3= *0.39*	-0.001	**-0.214**	0.013	0.005	**-0.233**	0.003	-0.001	**-0.228**	0.008	0.008

Note. CP: ceiling proportion. Unsatisfactory results are highlighted in bold.

Table S8. Moderated regression simulation results: Relative bias of regression coefficient β_2 for conditions in Scenario 1 where the outcome and the focal predictor have ceiling effects

		CP = 0%	CP = 10%			CP = 20%			CP = 30%		
		Reference	Conventional	Tobit ML	Tobit Bayesian	Conventional	Tobit ML	Tobit Bayesian	Conventional	Tobit ML	Tobit Bayesian
						$\rho = 0.3$					
N = 50	$\beta_3 = 0$	0.000	**-0.253**	0.008	0.047	**-0.348**	0.013	0.070	**-0.436**	0.009	0.096
	$\beta_3 = 0.39$	-0.005	**-0.331**	0.002	0.039	**-0.408**	0.006	0.064	**-0.468**	0.021	0.096
N = 100	$\beta_3 = 0$	-0.001	**-0.255**	0.001	0.022	**-0.356**	0.000	0.035	**-0.443**	-0.006	0.045
	$\beta_3 = 0.39$	-0.003	**-0.334**	-0.001	0.019	**-0.408**	0.008	0.035	**-0.477**	-0.003	0.036
N = 200	$\beta_3 = 0$	-0.001	**-0.260**	-0.002	0.012	**-0.356**	0.003	0.026	**-0.447**	-0.007	0.023
	$\beta_3 = 0.39$	0.000	**-0.339**	0.001	0.013	**-0.417**	-0.003	0.013	**-0.479**	0.001	0.024
N = 500	$\beta_3 = 0$	0.003	**-0.256**	0.002	0.011	**-0.358**	-0.003	0.013	**-0.442**	0.001	0.023
	$\beta_3 = 0.39$	-0.002	**-0.338**	0.000	0.007	**-0.419**	-0.001	0.010	**-0.480**	-0.003	0.011
						$\rho = 0$					
N = 50	$\beta_3 = 0$	0.003	**-0.229**	0.008	0.034	**-0.329**	0.014	0.055	**-0.427**	0.013	0.072
	$\beta_3 = 0.39$	-0.003	**-0.279**	0.005	0.032	**-0.367**	-0.006	0.031	**-0.426**	0.011	0.060
N = 100	$\beta_3 = 0$	-0.006	**-0.230**	0.006	0.018	**-0.336**	0.004	0.023	**-0.434**	-0.006	0.021
	$\beta_3 = 0.39$	-0.006	**-0.280**	0.009	0.021	**-0.363**	0.008	0.026	**-0.420**	0.009	0.032
N = 200	$\beta_3 = 0$	0.000	**-0.238**	-0.002	0.005	**-0.339**	0.000	0.009	**-0.427**	0.002	0.013
	$\beta_3 = 0.39$	0.002	**-0.284**	0.002	0.007	**-0.363**	0.003	0.011	**-0.425**	0.003	0.015
N = 500	$\beta_3 = 0$	0.000	**-0.238**	-0.003	0.000	**-0.340**	-0.003	0.001	**-0.430**	0.000	0.006
	$\beta_3 = 0.39$	0.000	**-0.282**	0.005	0.007	**-0.363**	0.000	0.004	**-0.429**	0.001	0.006

Note. CP: ceiling proportion; Unsatisfactory results are highlighted in bold.

Table S9. Moderated regression simulation results: Empirical coverage rates (%) of regression coefficient β_1 for conditions in Scenario 1 where the outcome and the focal predictor have ceiling effects

		CP = 0% Reference	CP = 10% Conventional	Tobit ML	Tobit Bayesian	CP = 20% Conventional	Tobit ML	Tobit Bayesian	CP = 30% Conventional	Tobit ML	Tobit Bayesian
						$\rho = 0.3$					
N = 50	$\beta_3 = 0$	92.7	**76.1**	94.2	94.4	**69.8**	95.8	96.7	**71.5**	93.4	94.0
	$\beta_3 = 0.39$	94.9	**60.0**	93.8	94.5	**53.7**	94.5	95.0	**55.6**	92.9	93.2
N = 100	$\beta_3 = 0$	94.9	**52.8**	93.9	94.7	**51.1**	94.1	94.6	**53.6**	92.7	93.7
	$\beta_3 = 0.39$	94.3	**30.3**	94.3	95.4	**25.4**	92.7	93.2	**31.8**	93.6	94.6
N = 200	$\beta_3 = 0$	93.7	**26.8**	94.4	94.7	**21.4**	94.4	94.8	**28.1**	93.2	95.6
	$\beta_3 = 0.39$	95.3	**6.0**	94.3	94.3	**5.2**	93.9	94.6	**9.3**	91.6	94.4
N = 500	$\beta_3 = 0$	96.0	**1.5**	95.4	96.1	**1.3**	94.6	94.2	**1.9**	92.0	94.4
	$\beta_3 = 0.39$	94.8	**0.1**	94.0	94.1	**0.0**	95.4	95.5	**0.1**	92.9	95.1
						$\rho = 0$					
N = 50	$\beta_3 = 0$	92.8	**79.4**	94.1	95.0	**78.3**	94.3	94.7	**75.6**	93.6	95.5
	$\beta_3 = 0.39$	91.6	**68.3**	93.3	94.9	**62.1**	94.0	94.8	**64.3**	94.5	96.2
N = 100	$\beta_3 = 0$	93.9	**65.8**	95.3	95.2	**60.1**	93.9	94.5	**63.4**	94.0	94.8
	$\beta_3 = 0.39$	93.4	**44.3**	93.6	94.2	**42.8**	92.8	93.7	**44.6**	94.0	95.0
N = 200	$\beta_3 = 0$	94.5	**37.8**	94.2	94.4	**35.0**	93.2	93.5	**39.6**	94.8	95.5
	$\beta_3 = 0.39$	94.8	**13.6**	94.7	95.1	**13.8**	93.9	94.1	**17.3**	93.1	95.3
N = 500	$\beta_3 = 0$	94.9	**5.9**	93.9	95.1	**3.3**	96.2	96.3	**8.4**	95.2	95.2
	$\beta_3 = 0.39$	94.0	**0.2**	94.3	95.1	**0.1**	94.4	94.7	**0.7**	95.0	95.9

Note. CP: ceiling proportion; Unsatisfactory results are highlighted in bold.

Table S10. Moderated regression simulation results: Empirical coverage rates (%) of regression coefficient β_2 for conditions in Scenario 1 where the outcome and the focal predictor have ceiling effects

		CP = 0%	CP = 10%			CP = 20%			CP = 30%		
		Reference	Conventional	Tobit ML	Tobit Bayesian	Conventional	Tobit ML	Tobit Bayesian	Conventional	Tobit ML	Tobit Bayesian
						$\rho = 0.3$					
N = 50	$\beta_3 = 0$	92.6	**48.1**	92.7	94.6	**20.2**	93.1	93.1	**7.6**	93.8	93.6
	$\beta_3 = 0.39$	92.7	**31.1**	94.4	95.7	**13.5**	94.7	95.6	**5.9**	94.0	94.5
N = 100	$\beta_3 = 0$	93.1	**19.7**	95.9	95.5	**1.9**	93.1	94.0	**0.3**	93.3	94.2
	$\beta_3 = 0.39$	95.1	**8.0**	94.4	94.3	**1.2**	95.4	95.8	**0.2**	93.1	94.6
N = 200	$\beta_3 = 0$	94.7	**2.5**	94.2	93.9	**0.0**	95.7	94.9	**0.0**	93.7	94.6
	$\beta_3 = 0.39$	94.9	**0.3**	95.1	95.5	**0.0**	94.2	94.7	**0.0**	93.4	94.8
N = 500	$\beta_3 = 0$	95.2	**0.1**	96.5	96.6	**0.0**	94.2	94.7	**0.0**	92.9	94.3
	$\beta_3 = 0.39$	95.8	**0.0**	94.8	95.0	**0.0**	94.8	95.1	**0.0**	95.1	95.5
						$\rho = 0$					
N = 50	$\beta_3 = 0$	93.9	**50.9**	92.8	94.2	**23.3**	93.2	94.2	**6.2**	93.2	94.0
	$\beta_3 = 0.39$	91.6	**40.2**	94.6	95.0	**16.5**	93.9	94.6	**8.0**	93.2	94.2
N = 100	$\beta_3 = 0$	93.1	**26.2**	94.4	95.4	**2.9**	93.7	94.9	**0.0**	95.7	96.0
	$\beta_3 = 0.39$	93.2	**13.8**	93.6	93.8	**2.9**	93.8	94.1	**0.3**	95.4	96.2
N = 200	$\beta_3 = 0$	94.8	**3.4**	95.0	95.1	**0.1**	94.5	95.2	**0.0**	94.7	94.8
	$\beta_3 = 0.39$	94.4	**1.2**	96.5	96.6	**0.0**	95.3	95.2	**0.0**	94.4	95.0
N = 500	$\beta_3 = 0$	94.5	**0.0**	95.6	95.8	**0.0**	95.8	95.7	**0.0**	95.4	95.2
	$\beta_3 = 0.39$	96.9	**0.0**	94.6	94.7	**0.0**	95.2	95.2	**0.0**	95.0	94.9

Note. CP: ceiling proportion; Unsatisfactory results are highlighted in bold.

Table S11. Moderated regression simulation results: Convergence rates (%) for conditions in Scenario 2 where the moderator has ceiling effects

		CP = 0%	CP = 10%			CP = 20%			CP = 30%		
		Reference	Conventional	Tobit ML	Tobit Bayesian	Conventional	Tobit ML	Tobit Bayesian	Conventional	Tobit ML	Tobit Bayesian
		$\rho = 0.3$									
N = 50	$\beta_3 = 0$	100.0	100.0	100.0	100.0	100.0	100.0	100.0	100.0	99.9	100.0
	$\beta_3 = 0.39$	100.0	100.0	100.0	100.0	100.0	100.0	100.0	100.0	99.8	100.0
N = 100	$\beta_3 = 0$	100.0	100.0	100.0	100.0	100.0	100.0	100.0	100.0	100.0	100.0
	$\beta_3 = 0.39$	100.0	100.0	100.0	100.0	100.0	100.0	100.0	100.0	100.0	100.0
N = 200	$\beta_3 = 0$	100.0	100.0	100.0	100.0	100.0	100.0	100.0	100.0	100.0	100.0
	$\beta_3 = 0.39$	100.0	100.0	100.0	100.0	100.0	100.0	100.0	100.0	100.0	100.0
N = 500	$\beta_3 = 0$	100.0	100.0	100.0	100.0	100.0	100.0	100.0	100.0	100.0	100.0
	$\beta_3 = 0.39$	100.0	100.0	100.0	100.0	100.0	100.0	100.0	100.0	100.0	100.0
		$\rho = 0$									
N = 50	$\beta_3 = 0$	100.0	100.0	100.0	100.0	100.0	100.0	100.0	100.0	99.9	100.0
	$\beta_3 = 0.39$	100.0	100.0	99.8	100.0	100.0	99.8	100.0	100.0	99.6	100.0
N = 100	$\beta_3 = 0$	100.0	100.0	100.0	100.0	100.0	100.0	100.0	100.0	100.0	100.0
	$\beta_3 = 0.39$	100.0	100.0	100.0	100.0	100.0	100.0	100.0	100.0	100.0	100.0
N = 200	$\beta_3 = 0$	100.0	100.0	100.0	100.0	100.0	100.0	100.0	100.0	100.0	100.0
	$\beta_3 = 0.39$	100.0	100.0	100.0	100.0	100.0	100.0	100.0	100.0	100.0	100.0
N = 500	$\beta_3 = 0$	100.0	100.0	100.0	100.0	100.0	100.0	100.0	100.0	100.0	100.0
	$\beta_3 = 0.39$	100.0	100.0	100.0	100.0	100.0	100.0	100.0	100.0	100.0	100.0

Note. CP: ceiling proportion; Unsatisfactory results are highlighted in bold.

Table S12. Moderated regression simulation results: Relative bias of regression coefficient β_1 for conditions in Scenario 2 where the moderator has ceiling effects

		CP = 0% Reference	CP = 10% Conventional	Tobit ML	Tobit Bayesian	CP = 20% Conventional	Tobit ML	Tobit Bayesian	CP = 30% Conventional	Tobit ML	Tobit Bayesian
		$\rho = 0.3$									
N = 50	$\beta_3 = 0$	-0.007	0.021	0.008	0.016	0.034	-0.001	0.016	0.057	-0.002	0.025
	$\beta_3 = 0.39$	-0.001	0.033	0.001	0.009	0.071	-0.010	0.006	**0.142**	-0.008	0.018
N = 100	$\beta_3 = 0$	0.001	0.000	-0.015	-0.007	0.036	0.002	0.019	0.060	-0.001	0.025
	$\beta_3 = 0.39$	0.002	0.039	0.004	0.012	0.077	-0.008	0.008	**0.158**	-0.002	0.025
N = 200	$\beta_3 = 0$	-0.002	0.009	-0.006	0.003	0.030	-0.004	0.012	0.060	-0.001	0.026
	$\beta_3 = 0.39$	0.002	0.039	0.002	0.010	0.088	-0.001	0.015	**0.160**	0.000	0.026
N = 500	$\beta_3 = 0$	-0.001	0.012	-0.003	0.005	0.032	-0.003	0.013	0.059	-0.002	0.024
	$\beta_3 = 0.39$	0.001	0.032	-0.006	0.003	0.089	-0.001	0.014	**0.157**	-0.004	0.021
		$\rho = 0$									
N = 50	$\beta_3 = 0$	0.003	0.004	0.003	0.003	0.008	0.009	0.009	-0.004	-0.004	-0.004
	$\beta_3 = 0.39$	0.002	0.021	0.004	0.004	0.046	0.000	0.000	0.088	-0.001	0.000
N = 100	$\beta_3 = 0$	-0.002	-0.002	-0.001	-0.001	0.004	0.003	0.003	0.000	-0.001	-0.001
	$\beta_3 = 0.39$	-0.003	0.019	0.001	0.001	0.051	0.003	0.003	0.090	0.001	0.000
N = 200	$\beta_3 = 0$	0.002	0.000	0.000	0.000	-0.002	-0.003	-0.002	0.001	0.001	0.001
	$\beta_3 = 0.39$	0.000	0.019	-0.001	-0.001	0.050	0.002	0.001	0.093	0.003	0.002
N = 500	$\beta_3 = 0$	0.000	-0.001	-0.001	-0.001	0.001	0.001	0.001	-0.002	-0.002	-0.002
	$\beta_3 = 0.39$	-0.001	0.021	0.001	0.001	0.049	0.000	0.000	0.092	0.001	0.000

Note. CP: ceiling proportion; Unsatisfactory results are highlighted in bold.

Table S13. Moderated regression simulation results: Relative bias of regression coefficient β_2 for conditions in Scenario 2 where the moderator has ceiling effects

		CP = 0% Reference	CP = 10% Conventional	Tobit ML	Tobit Bayesian	CP = 20% Conventional	Tobit ML	Tobit Bayesian	CP = 30% Conventional	Tobit ML	Tobit Bayesian
						$\rho = 0.3$					
N = 50	$\beta_3 = 0$	0.000	0.075	0.014	0.001	**0.154**	0.004	-0.010	**0.252**	0.008	-0.008
	$\beta_3 = 0.39$	-0.005	0.085	0.007	-0.005	**0.186**	0.013	0.002	**0.314**	0.031	0.021
N = 100	$\beta_3 = 0$	-0.001	0.076	0.012	-0.001	**0.159**	0.010	-0.004	**0.246**	0.008	-0.007
	$\beta_3 = 0.39$	-0.003	0.085	0.007	-0.003	**0.191**	0.017	0.007	**0.290**	0.012	0.003
N = 200	$\beta_3 = 0$	-0.001	0.078	0.013	0.001	**0.162**	0.014	0.000	**0.243**	0.006	-0.009
	$\beta_3 = 0.39$	0.000	0.092	0.012	0.002	**0.183**	0.011	0.002	**0.288**	0.012	0.004
N = 500	$\beta_3 = 0$	0.003	0.079	0.014	0.002	**0.158**	0.013	-0.001	**0.248**	0.010	-0.004
	$\beta_3 = 0.39$	-0.001	0.091	0.011	0.001	**0.185**	0.014	0.005	**0.290**	0.014	0.006
						$\rho = 0$					
N = 50	$\beta_3 = 0$	0.003	0.076	0.012	0.001	**0.165**	0.017	0.006	**0.258**	0.014	0.003
	$\beta_3 = 0.39$	-0.003	0.072	0.007	-0.004	**0.153**	0.007	-0.004	**0.253**	0.012	0.000
N = 100	$\beta_3 = 0$	-0.006	0.075	0.011	0.001	**0.155**	0.010	0.000	**0.239**	0.002	-0.008
	$\beta_3 = 0.39$	-0.006	0.079	0.013	0.003	**0.159**	0.011	0.001	**0.251**	0.012	0.001
N = 200	$\beta_3 = 0$	0.000	0.072	0.008	-0.002	**0.156**	0.010	0.000	**0.243**	0.011	0.001
	$\beta_3 = 0.39$	0.002	0.075	0.010	-0.001	**0.155**	0.010	0.000	**0.246**	0.011	0.001
N = 500	$\beta_3 = 0$	-0.002	0.072	0.007	-0.003	**0.155**	0.010	0.000	**0.241**	0.009	-0.001
	$\beta_3 = 0.39$	0.000	0.078	0.012	0.002	**0.154**	0.011	0.001	**0.243**	0.009	0.000

Note. CP: ceiling proportion; Unsatisfactory results are highlighted in bold.

Table S14. Moderated regression simulation results: Empirical bias and relative bias of regression coefficient β_3 for conditions in Scenario 2 where the moderator has ceiling effects

		CP = 0%	CP = 10%			CP = 20%			CP = 30%		
		Reference	Conventional	Tobit ML	Tobit Bayesian	Conventional	Tobit ML	Tobit Bayesian	Conventional	Tobit ML	Tobit Bayesian
						$\rho = 0.3$					
N = 50	$\beta_3 = 0$	-0.003	0.034	-0.006	0.003	0.081	0.007	0.027	0.115	0.007	0.038
	$\beta_3 = 0.39$	-0.004	**0.217**	0.035	0.052	**0.370**	0.017	0.065	**0.540**	0.006	0.085
N = 100	$\beta_3 = 0$	0.004	0.043	0.004	0.013	0.069	-0.002	0.017	0.098	0.000	0.029
	$\beta_3 = 0.39$	-0.011	**0.199**	0.020	0.036	**0.350**	0.007	0.050	**0.543**	0.019	0.092
N = 200	$\beta_3 = 0$	0.001	0.040	0.002	0.010	0.068	-0.001	0.017	0.097	0.001	0.029
	$\beta_3 = 0.39$	0.002	**0.199**	0.016	0.031	**0.345**	0.007	0.048	**0.519**	0.008	0.076
N = 500	$\beta_3 = 0$	-0.001	0.039	0.001	0.009	0.067	-0.001	0.017	0.098	0.003	0.031
	$\beta_3 = 0.39$	-0.007	**0.188**	0.010	0.024	**0.350**	0.014	0.053	**0.518**	0.014	0.079
						$\rho = 0$					
N = 50	$\beta_3 = 0$	0.002	0.003	0.003	0.003	0.000	0.000	0.000	0.004	0.003	0.003
	$\beta_3 = 0.39$	0.007	0.080	0.010	0.002	**0.173**	0.015	0.009	**0.265**	0.002	-0.001
N = 100	$\beta_3 = 0$	-0.002	-0.007	-0.006	-0.006	0.007	0.006	0.006	0.008	0.005	0.005
	$\beta_3 = 0.39$	-0.012	0.070	0.001	-0.007	**0.167**	0.016	0.007	**0.245**	0.008	-0.002
N = 200	$\beta_3 = 0$	-0.003	0.002	0.001	0.001	0.009	0.007	0.007	0.000	0.000	0.000
	$\beta_3 = 0.39$	0.008	0.081	0.014	0.005	**0.156**	0.011	0.001	**0.247**	0.016	0.004
N = 500	$\beta_3 = 0$	0.001	-0.001	-0.001	-0.001	0.002	0.001	0.001	-0.004	-0.003	-0.003
	$\beta_3 = 0.39$	-0.002	0.075	0.009	0.000	**0.151**	0.007	-0.003	**0.244**	0.011	-0.001

Note. CP: ceiling proportion. When $\beta_3 = 0$, the displayed values are empirical biases; when $\beta_3 = 0.39$, the displayed values are relative biases. Unsatisfactory results are highlighted in bold.

Table S15. Moderated regression simulation results: Empirical coverage rates (%) of regression coefficient β_1 for conditions in Scenario 2 where the moderator has ceiling effects

		CP = 0%	CP = 10%			CP = 20%			CP = 30%		
		Reference	Conventional	Tobit ML	Tobit Bayesian	Conventional	Tobit ML	Tobit Bayesian	Conventional	Tobit ML	Tobit Bayesian
		$\rho = 0.3$									
N = 50	$\beta_3 = 0$	92.7	92.3	92.8	94.5	94.6	95.2	96.4	92.5	94.0	94.9
	$\beta_3 = 0.39$	94.9	92.9	94.3	95.6	**89.6**	93.1	94.7	**82.4**	93.9	95.3
N = 100	$\beta_3 = 0$	94.9	94.4	94.2	95.1	93.3	94.2	94.8	92.0	95.1	95.1
	$\beta_3 = 0.39$	94.3	91.5	93.4	93.8	**86.3**	93.0	93.7	**70.6**	93.7	93.3
N = 200	$\beta_3 = 0$	93.7	95.0	95.5	95.5	91.6	95.1	94.4	**88.5**	96.0	94.8
	$\beta_3 = 0.39$	95.3	91.3	94.5	94.0	**78.5**	95.0	94.2	**52.6**	93.3	93.2
N = 500	$\beta_3 = 0$	96.0	94.6	95.1	94.9	**90.6**	93.8	93.3	**80.4**	95.3	93.1
	$\beta_3 = 0.39$	95.2	**88.3**	94.3	94.3	**57.8**	94.6	94.0	**16.7**	94.6	91.6
		$\rho = 0$									
N = 50	$\beta_3 = 0$	92.8	92.1	92.3	93.8	94.6	94.1	95.5	92.2	91.7	93.3
	$\beta_3 = 0.39$	91.6	93.7	94.6	96.1	92.5	94.6	96.3	**89.7**	94.4	95.9
N = 100	$\beta_3 = 0$	93.9	95.0	95.1	95.8	93.8	93.9	94.5	94.8	94.9	95.9
	$\beta_3 = 0.39$	93.4	94.1	93.9	94.8	91.4	93.0	93.7	**84.3**	93.8	94.1
N = 200	$\beta_3 = 0$	94.5	95.0	95.6	95.8	94.7	94.8	94.8	95.0	95.7	95.5
	$\beta_3 = 0.39$	94.8	94.1	95.0	95.3	**89.0**	95.5	95.9	**75.7**	95.7	95.8
N = 500	$\beta_3 = 0$	94.7	95.1	95.2	95.0	95.3	95.3	95.2	94.6	94.9	94.5
	$\beta_3 = 0.39$	94.0	91.9	94.1	94.2	**81.9**	95.2	95.1	**54.6**	95.1	95.1

Note. CP: ceiling proportion; Unsatisfactory results are highlighted in bold.

Table S16. Moderated regression simulation results: Empirical coverage rates (%) of regression coefficient β_2 for conditions in Scenario 2 where the moderator has ceiling effects

		CP = 0%	CP = 10%			CP = 20%			CP = 30%		
		Reference	Conventional	Tobit ML	Tobit Bayesian	Conventional	Tobit ML	Tobit Bayesian	Conventional	Tobit ML	Tobit Bayesian
						$\rho = 0.3$					
N = 50	$\beta_3 = 0$	92.6	91.6	93.9	95.2	**84.7**	93.7	95.1	**78.8**	93.7	95.1
	$\beta_3 = 0.39$	92.7	**88.9**	93.0	94.7	**82.5**	93.5	94.8	**71.8**	93.3	94.6
N = 100	$\beta_3 = 0$	93.1	**89.9**	94.0	95.3	**76.9**	94.1	94.9	**61.2**	92.5	93.7
	$\beta_3 = 0.39$	95.1	**87.0**	93.4	94.0	**69.9**	95.3	95.6	**55.0**	93.6	94.5
N = 200	$\beta_3 = 0$	94.7	**83.6**	93.2	94.1	**58.4**	95.0	95.3	**38.2**	95.8	95.7
	$\beta_3 = 0.39$	94.9	**81.6**	94.7	94.9	**51.6**	95.8	96.3	**26.1**	94.9	94.9
N = 500	$\beta_3 = 0$	95.2	**66.0**	95.5	96.0	**25.4**	95.1	95.4	**4.6**	92.6	95.0
	$\beta_3 = 0.39$	94.9	**58.5**	94.5	96.0	**11.8**	93.7	94.4	**1.0**	92.4	93.7
						$\rho = 0$					
N = 50	$\beta_3 = 0$	93.9	**90.2**	93.4	95.4	**83.7**	92.8	94.3	**74.7**	93.5	94.5
	$\beta_3 = 0.39$	91.6	91.2	93.3	95.0	**84.2**	93.1	95.0	**75.3**	93.3	95.1
N = 100	$\beta_3 = 0$	93.1	**90.2**	94.9	95.4	**76.2**	93.7	94.2	**62.1**	93.9	94.6
	$\beta_3 = 0.39$	93.2	**87.9**	94.9	95.3	**75.4**	94.6	95.0	**58.9**	95.3	95.8
N = 200	$\beta_3 = 0$	94.8	**85.0**	95.1	94.9	**59.1**	94.5	94.7	**31.1**	94.6	95.2
	$\beta_3 = 0.39$	94.4	**85.2**	95.1	95.3	**59.0**	94.8	95.1	**32.4**	96.2	96.7
N = 500	$\beta_3 = 0$	95.3	**68.6**	96.0	96.0	**21.7**	94.6	95.2	**3.1**	95.0	95.2
	$\beta_3 = 0.39$	96.9	**65.4**	93.8	94.5	**22.1**	92.9	94.0	**2.9**	95.1	95.3

Note. CP: ceiling proportion; Unsatisfactory results are highlighted in bold.

Table S17. Moderated regression simulation results: Empirical Type I error rates (%) and coverage rates (%) of regression coefficient β_3 for conditions in Scenario 2 where the moderator has ceiling effects

		CP = 0% Reference	CP = 10% Conventional	Tobit ML	Tobit Bayesian	CP = 20% Conventional	Tobit ML	Tobit Bayesian	CP = 30% Conventional	Tobit ML	Tobit Bayesian
						$\rho = 0.3$					
N = 50	$\beta_3 = 0$	6.4	**8.2**	6.7	5.6	**8.2**	5.7	4.7	**10.4**	7.1	5.5
	$\beta_3 = 0.39$	93.6	**87.9**	93.5	95.1	**85.5**	94.3	94.7	**81.9**	92.5	92.9
N = 100	$\beta_3 = 0$	5.3	**8.9**	5.5	4.8	**10.2**	6.4	5.5	**11.1**	5.7	6.8
	$\beta_3 = 0.39$	93.8	**86.9**	94.9	94.5	**80.7**	94.3	95.5	**70.6**	93.6	93.2
N = 200	$\beta_3 = 0$	5.4	**8.3**	5.6	5.2	**13.8**	5.9	6.2	**14.9**	5.8	5.8
	$\beta_3 = 0.39$	94.8	**80.2**	94.0	93.9	**67.5**	94.7	94.3	**50.6**	94.4	94.0
N = 500	$\beta_3 = 0$	4.6	**13.3**	5.3	5.6	**22.8**	5.1	6.8	**34.6**	4.2	**10.3**
	$\beta_3 = 0.39$	94.3	**69.2**	94.3	93.7	**35.1**	95.7	93.0	**13.1**	94.4	91.3
						$\rho = 0$					
N = 50	$\beta_3 = 0$	6.2	7.0	6.5	5.4	**7.9**	**8.1**	6.2	7.3	7.3	5.7
	$\beta_3 = 0.39$	93.0	94.3	95.0	96.3	92.2	93.9	95.2	**90.8**	93.7	95.3
N = 100	$\beta_3 = 0$	5.3	5.5	4.9	4.5	4.6	5.0	4.4	5.8	5.3	4.9
	$\beta_3 = 0.39$	94.6	92.0	94.7	95.2	91.0	94.7	95.2	**89.2**	94.5	95.6
N = 200	$\beta_3 = 0$	4.5	5.6	5.1	4.7	5.3	4.7	4.7	5.5	6.0	5.7
	$\beta_3 = 0.39$	95.7	91.7	94.4	94.6	**87.6**	93.6	93.4	**81.9**	93.8	93.8
N = 500	$\beta_3 = 0$	5.6	5.0	4.5	4.2	5.2	4.7	4.9	6.3	6.6	6.4
	$\beta_3 = 0.39$	95.6	91.0	95.5	95.5	**81.2**	96.0	95.5	**68.0**	96.8	96.4

Note. CP: ceiling proportion. When $\beta_3 = 0$, the displayed values are empirical Type I error rates; when $\beta_3 = 0.39$, the displayed values are coverage rates. Unsatisfactory results are highlighted in bold.

Pilot simulation study for evaluating the Tobit ML moderation regression model without integrating latent variable modeling

- Design: The ceiling proportions of the predictor and the outcome were set to 10%, 20% and 30%. The regression coefficients of β_1 and β_2 were fixed to be 0. Two β_3 values were considered: 0 and 0.39. Three sample sizes were considered: 100, 200, and 500.
- Mplus code for the Tobit ML moderation regression model without integrating latent variable modeling:

```
data: file is data.txt;
variable:
names are  x y z;
usevariables are x y z inter;
censored are x(a) y(a);
define:
inter=x*z;
analysis:
estimator = ML;
Model:
y on x z inter;
output:  CINTERVAL;
```

Table S18. Moderated regression simulation results from Tobit ML moderation regression model without integrating latent variable modeling: Empirical Bias, relative bias, empirical Type I error rates (%), and coverage rates (%) of regression coefficient β_3 for conditions in the pilot simulation study.

		CP = 0%	CP = 10%	CP = 20%	CP = 30%
		Empirical Bias or relative bias			
N = 100	$\beta_3 = 0$	0.002	0.003	-0.002	0.010
	$\beta_3 = 0.39$	0.034	**0.107**	**0.181**	**0.302**
N = 200	$\beta_3 = 0$	0.000	0.000	-0.004	0.000
	$\beta_3 = 0.39$	0.019	0.092	**0.175**	**0.269**
N = 500	$\beta_3 = 0$	-0.001	-0.002	0.001	0.002
	$\beta_3 = 0.39$	0.009	0.083	**0.164**	**0.272**
		Empirical Type I error rates or coverage rates			
N = 100	$\beta_3 = 0$	3.7	4.9	5.5	6.0
	$\beta_3 = 0.39$	92.9	93.7	91.7	**89.5**
N = 200	$\beta_3 = 0$	5.7	5.1	5.9	5.7
	$\beta_3 = 0.39$	93.6	93.5	**88.3**	**86.0**
N = 500	$\beta_3 = 0$	4.8	4.7	4.4	4.7
	$\beta_3 = 0.39$	95.4	**88.3**	**80.6**	**65.9**

Note. CP: ceiling proportion. When $\beta_3 = 0$, the displayed values are empirical bias or empirical Type I error rates; when $\beta_3 = 0.39$, the displayed values are relative bias or coverage rates. Unsatisfactory results are highlighted in bold.
